# Machine Learning for Multimodal Mental Health Detection: A Systematic Review of Passive Sensing Approaches

**DOI:** 10.3390/s24020348

**Published:** 2024-01-06

**Authors:** Lin Sze Khoo, Mei Kuan Lim, Chun Yong Chong, Roisin McNaney

**Affiliations:** 1 Department of Human-Centered Computing, Faculty of Information Technology, Monash University, Clayton, VIC 3800, Australia; roisin.mcnaney@monash.edu; 2 School of Information Technology, Monash University Malaysia, Subang Jaya 46150, Malaysia; lim.meikuan@monash.edu (M.K.L.); chong.chunyong@monash.edu (C.Y.C.)

**Keywords:** machine learning, mental health, multimodal detection, passive sensing, systematic review

## Abstract

As mental health (MH) disorders become increasingly prevalent, their multifaceted symptoms and comorbidities with other conditions introduce complexity to diagnosis, posing a risk of underdiagnosis. While machine learning (ML) has been explored to mitigate these challenges, we hypothesized that multiple data modalities support more comprehensive detection and that non-intrusive collection approaches better capture natural behaviors. To understand the current trends, we systematically reviewed 184 studies to assess feature extraction, feature fusion, and ML methodologies applied to detect MH disorders from passively sensed multimodal data, including audio and video recordings, social media, smartphones, and wearable devices. Our findings revealed varying correlations of modality-specific features in individualized contexts, potentially influenced by demographics and personalities. We also observed the growing adoption of neural network architectures for model-level fusion and as ML algorithms, which have demonstrated promising efficacy in handling high-dimensional features while modeling within and cross-modality relationships. This work provides future researchers with a clear taxonomy of methodological approaches to multimodal detection of MH disorders to inspire future methodological advancements. The comprehensive analysis also guides and supports future researchers in making informed decisions to select an optimal data source that aligns with specific use cases based on the MH disorder of interest.

## 1. Introduction

Mental health (MH) issues are pervasive in modern society, with the World Health Organization estimating that around 1 in 8, or 970 million people, were living with a mental health condition in 2019 [1]. The COVID-19 pandemic brought unprecedented times, leading to a reported increase in rates of anxiety and major depression by 25% in 2020 [2]. Subsequently, 42.9% of people in Australia aged between 16 and 85 years had experienced a mental disorder at some time in their lives as of 2022 [3], whereas 22.8% of adults in the U.S. were estimated to be experiencing mental illness as of 2021 [4]. With figures estimating that MH disorders will contribute to an economic loss of around USD 16 trillion globally by 2030 [5], it is unsurprising that MH has become a government priority worldwide. Specifically, the Comprehensive Mental Health Action Plan 2013–2030 [6] encompasses several global targets to promote improved mental health and well-being, where service coverage for MH conditions will have increased at least by half, and 80% of countries will have integrated mental health into primary health care by 2030. The impacts of MH issues on individuals’ lives are enormous. For example, people with mental illness reported having difficulty carrying out daily activities or requiring much energy and focus to meet demands at work [7], whereas those with depression experienced decreased enjoyment of activities and social interactions due to fluctuations in mood states [8]. Anxiety has also been found to reduce productivity and performance due to individuals’ attention being excessively directed towards other people’s perceptions [9]. In addition, research further demonstrated that emotional dysregulation introduces susceptibility to physical illnesses such as cardiovascular disease, viral infection, and immunodeficiency [10].

Despite the prevalence, several shortcomings exist in the current diagnosis and treatment of mental health disorders. These include comorbidities with other conditions that introduce complexity to diagnosis [11], the subsequent failure of clinicians to make accurate diagnoses due to obscurities of overlapping symptoms [11], the reliance on patients’ subjective recollection of behaviors [12], and the shortage of human resources available for mental health care [13]. The limitations above contribute to underdiagnosis, preventing people in need from receiving proper treatment. In light of the need to promote more accurate detection of MH disorders, researchers began exploring the application of artificial intelligence and machine learning (ML) in this domain. Such efforts are motivated by the ability of ML to analyze large amounts of data [14], distinguish data features [15], learn meaningful relationships between data [16], and apply the identified associations to make predictions about new data [17]. Coupling ML methods with qualitative analysis, visualization, and other interpretation tools further enhances the understanding of ML outputs [17], which can support clinical decisions and improve the comprehension of causes of specific MH disorders.

Existing research has seen numerous attempts to incorporate ML in healthcare, where effective ML methods can offer automation to harness large amounts of real-time data to improve the quality of patient care [18]. Nevertheless, the dynamic nature of an individual’s health, influenced by factors such as genetics, medical history, and lifestyle, remains a complex and demanding challenge to resolve [18]. Similarly, diagnoses of MH disorders are intricate due to the multifaceted nature of MH, involving emotional (e.g., sadness, helplessness), behavioral (e.g., isolation, self-talk), and physical (e.g., body aches, sleeplessness) aspects [19]. In addition, various perceived causes could contribute to MH issues, encompassing psychological (e.g., low self-esteem, overthinking), socioeconomic (e.g., racial and ethnic discrimination, poverty), and social (e.g., family conflicts, interpersonal relationships) factors [19]. As such, we hypothesize the need for multimodal data, i.e., data with multiple modalities each referring to a form of data or a signal from a data source, to achieve complementary effects for improved detection. For example, an existing work [20] has seen multimodal social media data, consisting of text, images, post metadata (e.g., time posted, likes, comments), and user metadata (e.g., profile description and image, followers), to offer additive effect when information from all modalities are incorporated. Additionally, the reliance of ML systems on extensive data and the heterogeneity of data from various sources necessitates the exploration of scalable and sophisticated ML methodologies to manage and standardize such big data, with considerations of privacy and security to ensure the confidentiality of patients’ information [21].

The pipeline of ML methodologies on multimodal data includes feature extraction for each modality, transformation and fusion of modality-specific features of various structures and dimensions and ML algorithms to learn from fused representations. Our preliminary investigation of recent surveys of ML applications to multimodal MH detection revealed several data sources, such as social media [22,23,24,25], smartphones [26,27], and wearable devices [27,28]. Nevertheless, we observed a limited evaluation of the current state of knowledge in each methodological phase mentioned above, in which the understanding is crucial to inform advancements in ML approaches. In summary, the gaps we identified are the need for (1) more effective ML approaches to reduce the risk of underdiagnosis and (2) ML methodologies for handling heterogeneous and extensive multimodal data to support the detection of multifaceted MH disorders.

In this systematic literature review (SLR), we address these limitations by analyzing individual methodological phases in greater detail. We further narrow our scope to studies adopting passive sensing, which gathers users’ data non-intrusively via ubiquitous sensors or devices and requires minimal user inputs. This decision is supported by our hypothesis that people’s natural behaviors are best captured when their daily routines are subject to the least possible obstructions [29,30]. Less intrusive approaches have also been shown to have better acceptance among the general population, with the need to carry/wear dedicated equipment being reported as off-putting and causing levels of discomfort [12,31]. From a recent survey [17], we learned that two key motivations for ML applications to mental health are the accessibility to behavioral data enabled by continuous and non-invasive approaches and the efficiency and cost-effectiveness of timely and automated data processing. Drawing inspiration from the survey above, we establish several criteria that we anticipate in data collection approaches that are practical to promote subsequent effective detection of MH disorders: (1) reliability (i.e., ensuring that the data closely represents actual behaviors), (2) verifiable ground truth, (3) cost-effectiveness, and (4) acceptability among the general population. Consequently, we conduct a detailed analysis of each data source based on these criteria. This SLR aims to (1) assess the current trend of multimodal ML approaches for detecting various MH disorders and (2) identify an optimal strategy leveraging passively sensed multimodal data and ML algorithms. Specifically, the research questions (RQs) we aim to address throughout our study are:

- RQ1—Which sources of passive sensing data are most effective for supporting the detection of MH disorders?
- RQ2—Which data fusion approaches are most effective for combining data features of varying modalities to prepare for training ML models to detect MH disorders?
- RQ3—What ML approaches have previous researchers used to successfully detect MH disorders from multimodal data?

The SLR is structured as follows: Section 2 outlines the research methods adopted in this review, followed by Section 3, which presents results that analyze the individual phases of existing methodologies, including data sources, feature extraction, modality fusion techniques, and the ML algorithms adopted. Based on the analysis, Section 4 then synthesizes the findings to address each RQ mentioned above and draws insights into recommendations and considerations for future researchers wishing to innovate in this space. Lastly, Section 5 concludes the study.

## 2. Materials and Methods

This section presents the review protocol for our SLR based on the PRISMA 2020 Statement [32], a guideline for healthcare-related studies [33] established based on the PRISMA 2009 Statement [34], and the Guidelines for SLRs in Software Engineering [35] published in 2007.

### 2.1. Search Strategy

We performed an exhaustive search on four online databases: Scopus, PubMed, ACM Digital Library, and IEEE Xplore. We chose these databases due to the abundance of published papers on the topic of concern and to represent the multidisciplinarity of the topic by having a diversity of papers across the fields of clinical science and computing science. As previously explained, we concentrate on studies utilizing data of at least two different modalities collected using ubiquitous devices and applying ML techniques for detecting MH disorders. Inspired by Zhang et al.’s [36] search strategy, we systematically constructed our search query based on aspects shown in Table 1.

We queried the databases by combining keywords within the same category with an OR operator and those across categories with an AND operator. We also considered different terminology variants by using wildcards (*), for instance, “well*” in our query string, because the term “wellbeing” may be spelled as “well-being” in certain studies. An example of our query string on Scopus is as follows:

“ALL (mental AND (health OR disorder OR illness OR well*)) AND TITLE-ABS-KEY (“artificial intelligence” OR “machine learning” OR model) AND TITLE-ABS-KEY (detect* OR predict* OR classif* OR monitor* OR recogn* OR identif*) AND TITLE-ABS-KEY (“social media” OR text* OR audio* OR speech* OR voice OR visual OR imag* OR video* OR smartphone* OR mobile OR wearable* OR sens*) AND PUBYEAR > 2014”.

We decided on the cutoff publication year of 2015 due to the consideration of the developmental trajectory of the research domain. Our preliminary observation revealed 2015 as a potential juncture where relevant studies began gaining momentum, coinciding with the introduction of AVEC 2013 [37] and AVEC 2014 [38] challenges focusing on facial expressions and vocal cues relating to specific MH conditions such as depression. We intended to ensure that our review encompasses more recent advancements for a comprehensive understanding of the field’s current state. In addition, the rapid development of technologies may render techniques from older publications obsolete or less relevant. Likewise, the relevance of findings related to smartphones and wearable devices may have evolved due to changes in their adoption among the general population over time.

### 2.2. Inclusion and Exclusion Criteria

To ensure the selection of studies that align with our research focus, we considered a study to be relevant if it fulfilled all of the following inclusion criteria:

- The study collects data passively via ubiquitous or wearable devices, considering the cost-effectiveness and general accessibility.
- The data is human generated, i.e., derived from individuals’ actions in an environment or interactions with specific platforms or devices.
- The data source involves at least two different modalities.
- The study adopts ML algorithms intending to detect one or more MH disorders.
- The study is written in English.
- The study was published from the year 2015 onwards (further details in the following section).

We excluded a study if any of the following exclusion criteria were satisfied:

- The study investigates data sources of a single modality or exclusively focuses on a specific modality, e.g., text-based approaches.
- The study specifically targets the pediatric population, i.e., toddlers and children below ten years old, as defined within the suggested adolescent age range of 10–24 years [39].
- The study targets a particular symptom of specific MH disorders, e.g., low mood, which is a common sign of depression.
- Data collection requires dedicated equipment or authorized resources:
  - Brain neuroimaging data, e.g., functional magnetic resonance imaging (fMRI), structural MRI (sMRI), electroencephalogram (EEG), electromyography (EMG), and photoplethysmography (PPG) signals
  - Clinical data, e.g., electronic health records (EHRs) and clinical notes
  - Genomic data
  - Body motions collected using specialized motion capture platforms or motor sensors
  - Makes use of Augmented Augmented Reality (AR) or Virtual Reality (VR) technology
- The study does not employ ML algorithms for detection/prediction, e.g., focusing on correlation/association analysis, treatment/intervention strategies, or proposing study protocols.
- The study is a survey, book, conference proceeding, workshop, or magazine
- The study is unpublished or non-peer-reviewed.

Since our work explicitly emphasizes multimodality to observe cross-modality fusion and interactions, we excluded studies emphasizing a single modality. For example, we do not consider those solely analyzing textual content from social media sources (e.g., Twitter) without incorporating broader online social behaviors, such as posting time distribution and interactions with other users through retweets and comments. Additionally, we omitted studies involving children, as it is well-established that factors, manifestations, and responses to MH conditions can differ significantly between children and adults [40]. Children may also often rely on parents and family environment for care and treatment [41,42].

While changes in MH states such as affect, emotion, and stress may serve as potential indicators of MH disorders such as depression and anxiety [43], it is noteworthy that these factors, when considered in isolation, do not necessarily equate to a complete MH diagnosis [44,45]. Therefore, we refined our focus by excluding studies that solely investigated these states. Due to practicality concerns, we also enforced the utilization of ubiquitous devices in data collection to ensure these tools are easily accessible and cost-effective.

### 2.3. Selection Process

**Figure 1:**
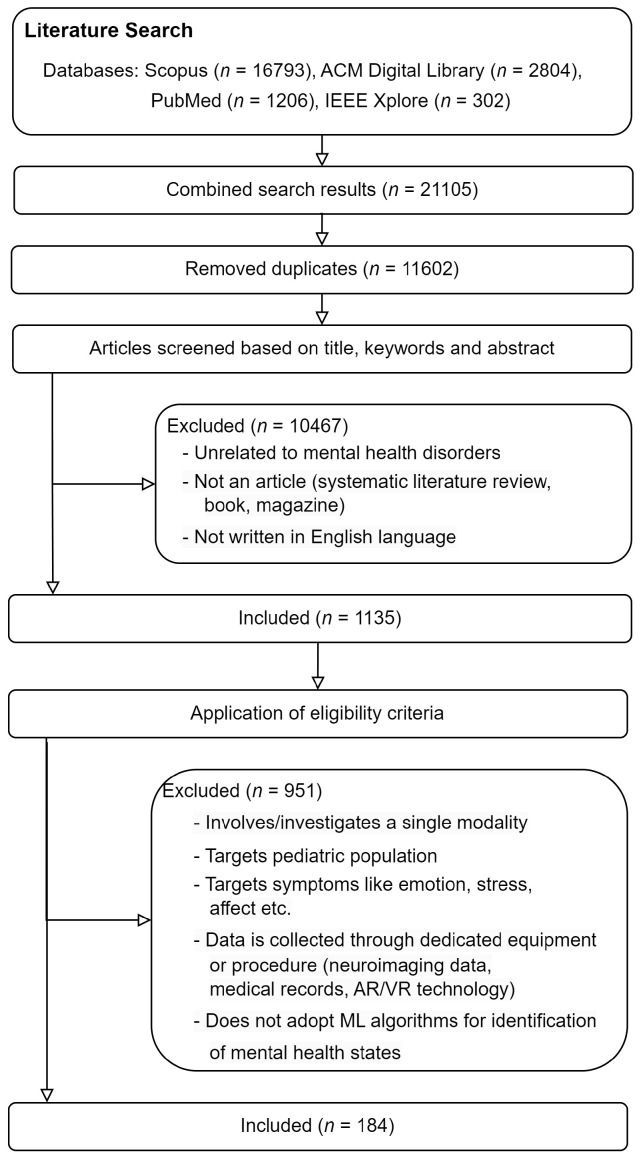
Flow diagram of study selection.

Figure 1 shows the literature search process as a flow diagram adapted from an example in the PRISMA guideline (https://www.bmj.com/content/bmj/339/bmj.b2700/F2.large.jpg (accessed on 19 September 2023)). After querying the selected databases, we re-evaluated the title, abstract, and keywords of individual studies to refine the results and remove duplicates. Subsequently, we manually applied the eligibility criteria to determine relevant studies for data extraction.

### 2.4. Data Extraction

Table 2 shows the information we extracted from individual studies and the corresponding mapping to the relevant research questions (RQs) where applicable.

### 2.5. Quality Assessment

We adapted a suggested checklist [35] to develop quality assessment criteria, shown in full in Table 3, that assigns a score to each study.

Most scoring, except for QC3, QC9, and QC13, adopt a three-item scale, satisfies = 1, does not satisfy = 0, and partially satisfies = 0.5, to evaluate whether a study complies with the corresponding criteria. The final quality score would be the summation of the score corresponding to the conformity of the checklist items. Acknowledging that healthy controls are not always necessary in relevant studies, we have specifically included checklist item QC3 due to our interest in the effectiveness of data sources and methodological approaches in distinguishing between individuals with and without MH disorders. Understanding general patterns in healthy controls also serves as a baseline for benchmarking to justify the significance of future findings related to those with MH conditions.

## 3. Results

**Figure 2:**
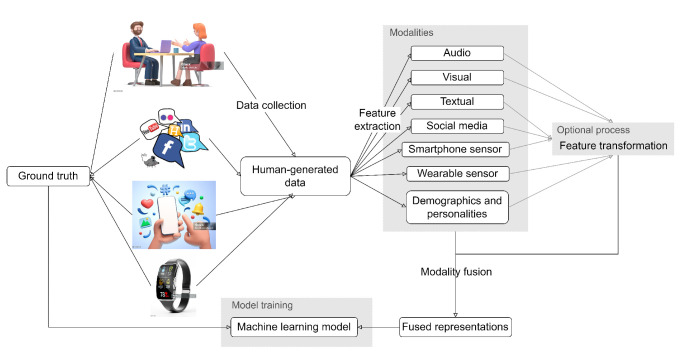
Pipeline of methodological phases involved in data extraction [224,225,226,227].

This section summarizes and analyzes the results we extracted from 184 relevant studies published from January 2015 to August 2023 based on Table 2. Table 4 displays the combinations of MH conditions investigated and the categories of data sources involved in all selected studies. Figure 2 shows the methodological pipeline involved in our data extraction. The following subsections describe and explain each extracted finding in detail.

**Table 4:** A compilation of relevant studies for data extraction.

Mental Health Conditions	Data Source
Depression	AV [43,46,47,48,49,50,51,52,53,54,55,56,57,58,59,60,61,62,63,64,65,66,67,68,69,70,71,72,73,74,75,76,77,78,79,80,81,82,83,84,85,86,87,88,89,90,91,92,93,94,95,96,97,98,99,100,101,102,103,104,105,106,107,108]
	SM [20,25,98,109,110,111,112,113,114,115,116,117,118,119,120,121,122,123,124,125,126,127,128,129,130,131,132,133,134,135,136,137,138,139,140,141,142,143,144,145,146,147,148]
	SS [99,100,104,105,149,150,151,152,153,154,155,156,157,158,159,160,161,162,163,164,165,166,167,168,169,170,171,172,173,174,175,176,177]
	WS [149,150,151,155,156,157,158,164,169,171,172,173,178,179,180,181,182]
Suicidal intent	AV [100,183,184]
	SM [185,186,187,188,189]
	SS [100,147,190]
	WS [181,182,191]
Bipolar disorder	AV [101,102,103,192,193,194,195,196,197,198,199,200]
	SM [201]
	SS [12,172,200,202]
	WS [172]
Schizophrenia	AV [203]
	SM [201,204]
	SS [172,205,206,207,208,209]
	WS [172,210]
Anxiety	AV [104,105,108,211]
	SM [148]
	SS [104,105,173,174,175,176,212]
	WS [173,179,213]
Autism spectrum disorder	AV [214]
	WS [215]
PTSD	AV [107,216]
	WS [216]
Eating disorder	SM [217,218,219]
Mental illness	SM [220]
Mental wellbeing	AV [221]
	SS [222,223]

AV: Audio and video recordings, SM: Social media, SS: Smartphone sensors, WS: Wearable sensors.

### 3.1. Data Source

The primary categories of data sources are (1) audio and video recordings (*n* = 82), (2) social media (*n* = 55), (3) smartphones (*n* = 54), and (4) wearable devices (*n* = 28).

#### 3.1.1. Audio and Video Recordings

Audio and video recordings of individuals were captured using video cameras, webcams, or microphones while they responded to interview questions or completed predetermined tasks in person or online. For example, Gratch et al. [228] conducted semi-structured interviews with individual participants with both neutral questions and those related to depression or PTSD events, which the authors recorded using a camera and close-talking microphone. In contrast, NEMSI (NEurological and Mental health Screening Instrument) [229] was proposed as a cloud-based system that automates data capture and the subsequent audio-visual processing for feature extraction and visualization. Before commencing the interviews, researchers [228,230] ensured that participants signed consent forms to collect highly identifiable recording data and share their data for research purposes. The researchers also offered transparency regarding the purpose of their study and data collection before participants provided their consent.

#### 3.1.2. Social Media

Meanwhile, social media platforms like Twitter, Reddit, Sina Microblog, Instagram, Facebook, YouTube, Flickr, and Blued offer a safe space for information sharing, communication, and expressing emotions. Various forms of user-generated content publicly available on these platforms are texts, images, social interactions (likes, comments, mentions, and shares), and user profile information (followers, followings, bio descriptions, profile images). Researchers could crawl content from these platforms using the provided application programming interface (API) by strategically querying content posted within a predetermined duration for observation, locating the presence of relevant phrases or keywords within textual content, or sourcing directly from discussion space revolving around specific MH conditions where applicable. For instance, Shen et al. [20] identified candidate social media users based on tweets containing the character string “depress” and utilized such tweets as anchor points to sample remaining tweets posted by the corresponding users within a month relative to anchor tweets. Meanwhile, Mishra et al. [185] scraped the top 100 posts from the “r/suicidalthoughts”, “r/suicidewatch”, and “takethislife.com” forums with an abundance of posts related to suicidal ideation.

Nevertheless, we observed limited ethical considerations and explicit mentions in existing studies regarding obtaining participants’ consent for utilizing their data for research purposes. For example, Yates et al. [231] discussed the privacy risks with posts crawled from Reddit as minimal since this data is publicly available on the platform. The researchers also described their privacy measures for ensuring that annotators and other researchers were only allowed access to anonymized posts after agreeing to adhere to the ethical guidelines for not attempting to contact or deanonymize data samples.

#### 3.1.3. Smartphones

Smartphone sensors, such as accelerometers, GPS, light sensors, and microphones, could collect and infer information about smartphone usage, physical activity, location, and an individual’s environment. Researchers have adopted existing mobile applications that collect sensing data, such as Purple Robot [232] (Android only), SensusMobile [99] (Android only), and LifeRhythm [233] (Android and iOS), and those with additional features, including Behavidence https://www.behavidence.com/ (accessed on 10 December 2023) (Android application that displays similarity scores of inferred behaviors to specific MH disorders), Insights [234] (Android application with customizable questionnaires), MoodMirror [235] (Chinese Android application that connects with a wristband via Bluetooth), or BiAffect https://www.biaffect.com/ (accessed on 10 December 2023) (iOS only), that collect keyboard typing data specifically. In contrast, some researchers developed mobile applications for their use cases using frameworks like AWARE [236] (collects sensor data from Android and iOS devices and supports integration with data analysis pipeline). These mobile applications act as a central management system, either storing data locally in individuals’ devices or transmitting them to a central server for processing and analysis.

Prior to data collection, researchers obtained participants’ consent and provided details about the data to be collected. Some researchers additionally conducted onboarding sessions for installing mobile applications, offered tutorials to operate them, and provided technical support throughout the data gathering duration [237]. Privacy measures were also implemented to minimize identifiability and the risks of data leakage during transmission, such as anonymizing participants, hashing phone calls and text messaging logs [237], and employing secure transmission protocols like HTTPS and SSL.

#### 3.1.4. Wearable Devices

Wearable devices have further enabled the collection of physical activity, movement, sleep, and physiological signals like heart rate (HR), electrodermal activity (EDA), skin temperature (ST), and galvanic skin response (GSR). Some examples of wearables are Empatica E4 wristbands [149,172,178], Microsoft Band 2 [150], Fitbit Charge or Flex trackers [151,155,164,180,181,182,191], and the Galaxy S3 smartwatch [169]. Data gathered through these devices were transmitted directly to an internet-connected server [215] or transferred via Bluetooth [210] to dedicated mobile applications that handle the transmission as described above. As such, existing studies executed similar procedures for obtaining participants’ consent before data collection and privacy measures to ensure secure data transmission.

Table 5 describes publicly available datasets discovered or released by studies included in this work for multimodal detection.

### 3.2. Data Ground Truth

Data used for supervised learning must have a ground truth (i.e., if the person to whom the data belong suffers from a specific MH disorder) so that ML models learn to distinguish data points of different ground-truth labels. The means of ground truth acquisition are (1) clinical assessment by trained psychiatrists or healthcare professionals and (2) self-reports by people themselves.

#### 3.2.1. Clinical Assessments

During clinical diagnoses, trained psychiatrists use clinically validated assessment scales with known symptoms of specific MH disorders to prompt patients to share their experiences. Establishing ground-truth knowledge varies based on experimental design in existing studies, where trained healthcare professionals could conduct clinical assessments before the data collection procedure and during other intermediate phases deemed necessary. For example, Grünerbl et al.’s [12] study involved psychologists conducting examinations every three weeks over the phone, using standard scale tests such as the Hamilton Rating Scale for Depression (HAMD) or Young Mania Rating Scale (YMRS), whereas participants were scheduled for monthly face-to-face clinical assessments with clinicians using the 7-item Brief Psychiatric Rating Scale (BPRS) in Wang et al.’s [206] study. On the other hand, participants could be recruited from the MH service within a hospital setting, where existing diagnoses of specific MH conditions are known, and clinical assessments could be reconducted during follow-ups and after discharge using the YMRS [230].

If access to healthcare professionals is unavailable, these scales can be administered through mobile applications or other devices to be answered and self-reported by subjects. Examples of scales used in both clinical and self-reported assessments are the Hamilton Depression Rating Scale (HDRS) [254], Patient Health Questionnaire-9 (PHQ-9) [255], Beck Depression Inventory (BDI) [256], and Center for Epidemiological Studies Depression Scale (CES-D) [257]. Researchers can compile and analyze the responses to derive ground truth based on established guidelines. For example, the summation score of the PHQ-9 scale corresponds to depression severity levels, where 5, 10, 15, and 20 represent mild, moderate, moderately severe, and severe depression, respectively [258].

#### 3.2.2. Self-Reports

In most cases where social media data has been scraped from public-facing platforms via application programming interfaces, users are not reachable due to security and privacy protection. As such, their MH states are not immediately acquirable since they do not usually disclose invasive information like medical history. Researchers have relied on textual or visual cues in users’ public posts to locate the existence of MH disorders for the purposes of ground truth. They detected self-reports where users explicitly disclosed being diagnosed with a specific MH disorder in their public posts by looking for sentence structure such as “I am diagnosed with…” [20].

This ground-truth acquisition method heavily relies on individuals’ willingness and openness to share content publicly on social media platforms. Therefore, to enhance the accuracy of ground truth labels, studies incorporated clinical opinions when annotating and labeling social media data. These opinions were sourced from trained psychiatrists or psychologists [112,131,145,186,219], as well as staff and students within the university settings with backgrounds in psychology [142,185]. For example, Abuhassan et al. [218] incorporated opinions from domain experts with specific expertise in eating disorders (EDs), psychology, mental health, and social media. The authors obtained a comprehensive and well-rounded annotation strategy to guide the categorization of social media users into individuals with an explicit diagnosis of EDs, healthcare professionals, communicators (i.e., those who communicate, exchange, and distribute information to the public), and non-ED individuals. The approaches above attempted to address the possibility of researchers overlooking implicit indicators of specific MH disorders or lacking sufficient clinical knowledge to make accurate inferences based on several posts created by each individual [135,189]. However, these efforts may not suffice, given that public content posted by individuals might be adapted with considerations of self-presentation factors.

### 3.3. Modality and Features

A range of modality-specific features within the datasets analyzed by researchers were found to support the identification of MH-related features in study participants. Table 6 provides a summary of these features and their findings relevant to MH diagnosis. See Appendix A for a more extensive view of the features and the corresponding extraction tools.

#### 3.3.1. Audio

Several popular approaches to extract audio features include adopting OpenSmile [267] to extract low-level descriptors (LLDs) and employing pre-trained deep learning (DL) models to extract high-level deep representations from either audio samples directly or transformed spectrogram images [66]. Researchers have identified several audio features to be significant indicators of MH conditions. For instance, Yang et al. [193] discovered histogram-based audio LLDs to be more effective than visual features in identifying bipolar disorder, and such indicators are more prominent in male samples. Meanwhile, from specific features such as energy contours, kurtosis, skewness, voiced tilt, energy entropy, and MFCCs, Belouali et al. [184] demonstrated that individuals with suicidal intent spoke using a less animated voice with flatter energy distribution and fewer bursts. Their speech had less vocal energy and less abrupt changes and were more monotonous. Other audio features found to be significant indicators of depression and PTSD include audio intensity, pitch, and spectral decrease [54,107]. Since it is beyond the scope of the current work to dive deep into audio samples and features, we direct interested researchers to an existing work [268] for greater details on audio processing and features that could be extracted at varying domains (e.g., time, frequency, and cepstrum) [54].

#### 3.3.2. Visual

Visual features were extracted by first locating individuals or objects in a video frame or a static image, identifying the corresponding feature points (e.g., facial landmarks, FAUs, upper body points), and then generating features using image processing tools including OpenFace [269], OpenCV [270], and OpenPose [271]. Pre-trained models could also be applied directly to visual samples to extract feature representations. For image frames extracted from video recordings, researchers could further capture dynamic aspects and transitions across a video, such as computing the speed and range of displacements of specific feature points between successive video frames and the variation across the entire video.

**Figure 3:**
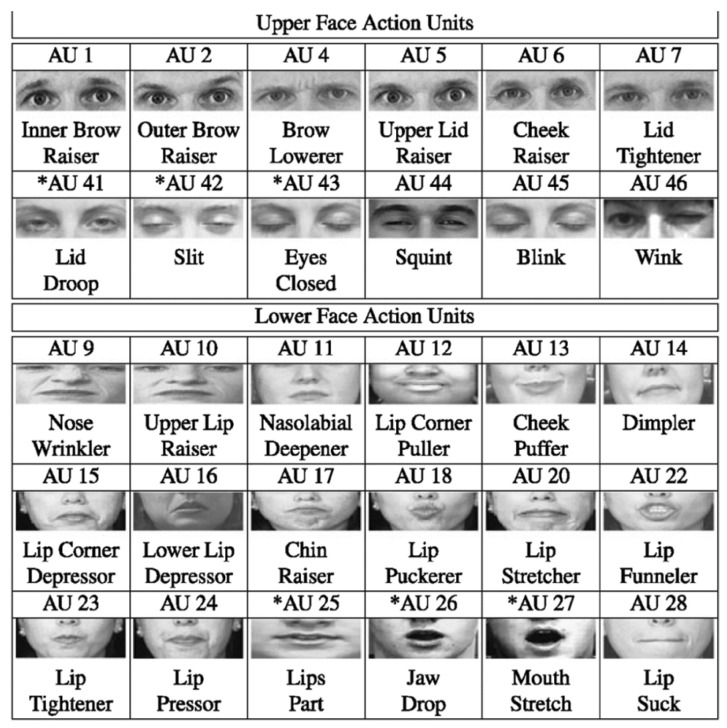
Examples of facial movements coded using facial action units [274].

**Figure 4:**
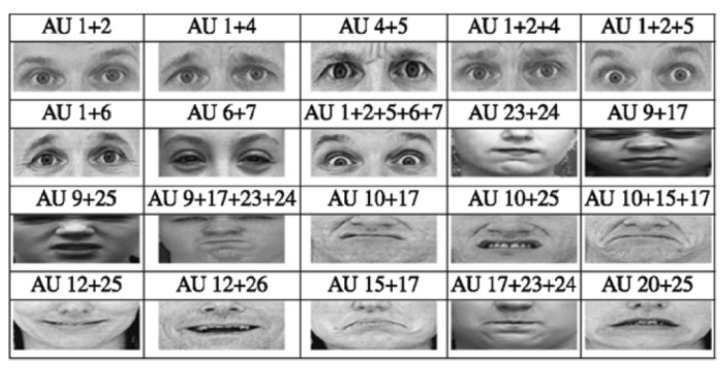
Examples of facial expressions resulting from combinations of facial action units [274].

Facial action units (FAUs) were introduced to describe facial movements [272], where each AU corresponds to contractions of specific facial muscles (e.g., AU5 represents raised upper eyelids, AU6 represents raised cheeks, and AU15 represents pulled-down lip corners [273] as shown in Figure 3). FAUs have shown significant promise in encoding facial expressions, each constituted by a combination of AUs [273] as shown in Figure 4. In the current context, Thati et al. [99] demonstrated that a few AUs correlate significantly with depressive symptoms, specifically, AU12, AU10, and AU25, corresponding to pulled lip corners, raised upper lips, and parted lips, respectively. Referring to both Figure 3 and Figure 4, this finding could be associated with a smiling expression comprising AU12 and AU25 and low mood as demonstrated by AU10. It could potentially indicate the “smiling depression” scenario mentioned by Ghosh et al. [132], where individuals with depression may choose to post more happy images compared to healthy controls who expressed diverse emotions.

In addition, facial appearance and emotions in shared images were significantly indicative of depression and PTSD [107,116]. While a few studies [25,132,220] showed that individuals with depression have lower tendencies to disclose facial identity, Gui et al. [111] found that they are more likely to post images with faces but of a lower average face count per image. From the revelation of more images of animals and health objects from Twitter and Reddit content, Uban et al. [128] hypothesized the possibility of individuals’ online help-seeking through viewing animal-related content that might improve psychological and emotional conditions and looking up causes of health events, diseases, and treatment options.

Meanwhile, other research studies [25,111,220] revealed that individuals with mental illness and depression post less colorful images of darker and grayer colors on social media compared to healthy controls, who prefer brighter and more vivid colors such as blue and green. These patterns potentially align with existing knowledge [275] regarding the influence of individuals’ mood on color preferences, where principal hues (e.g., red, yellow) and intermediate hues (e.g., yellow-red, blue-green) evoked higher positive emotions than achromatic colors like black and white. Specifically, Yazdavar et al. [25] demonstrated a strong positive correlation between self-reported depressive symptoms and individuals’ tendency to perceive surroundings as grey or lacking colors. In contrast, Xu et al. [220] further computed pleasure, arousal, and dominance scores from brightness and saturation values. The authors then discovered that individuals with mental illness preferred less saturated images (i.e., containing more grey [276]), which implied higher dominance and arousal than healthy controls.

#### 3.3.3. Textual

In addition to textual content written by individuals, researchers also obtained textual transcripts from audio samples using speech-to-text tools on Google Cloud Platform, AWS Transcribe [277], or Transcriber-AG https://transag.sourceforge.net/ (accessed on 10 December 2023). Tools like Linguistic Inquiry and Word Count (LIWC) [278], Suite of Automatic Linguistic Analysis Tools (SALAT) [279], and Natural Language Toolkit (NLTK) [280] were adopted on textual content to identify nouns, adjectives, pronouns, or specific words referring to social processes and psychological states, where linguistic features were generated as the occurrence of words in specific categories. Meanwhile, sentiment-related features like sentiment polarity scores were obtained from sentiment analysis tools, including Stanford NLP toolkit [281], Sentiment Analysis and Cognition Engine (SEANCE) [282], and Affective Norms for English Words ratings (ANEW) [283]. High-level textual representations could also be obtained via language models, such as BERT [266], Paragraph Vector (PV) [284], and XLNet [285], to represent each word using a vector. The overall textual representations could be obtained via concatenating directly, averaging, or applying attention mechanisms on word-level representations to emphasize more significant features.

Abundant studies consistently highlighted the prominent correlations between textual features and MH conditions. For example, the significance of linguistic features in MH identification was accentuated by compelling evidence showing that individuals with depression and suicidal intent used more first-person pronouns, possibly reflecting their suppressed nature. This linguistic pattern was observed in textual content across various social media platforms, including Weibo [109,123], Instagram [116], Twitter [25,118,121], and Reddit [186], as well as in transcribed audio recordings [184]. Meanwhile, several other studies [123,187] further found more frequent usage of the word “others” or third-person pronouns (e.g., “they”, “them”, “he”, “she”) than healthy controls, which the authors hypothesized as the tendency of depressive or suicidal individuals in acquiring physiological distance and reluctant to show feelings. In addition, researchers found individuals with depression, suicidal intent, and schizophrenia exhibiting a pronounced expression of negative emotions compared to healthy controls. This observation is substantiated by various features, including the frequency of negative words [20,118,123,204,220] and negative emoticons [130,188], as well as negative sentiment scores of overall sentences [121].

In contrast, specific keywords could be indicative, such as references to personal events like “work pressure”, “divorce”, and “break up” [118]; biological processes like “eat”, “blood”, and “pain” [116]; or family references like “daughter”, “dad”, and “aunt” [184]. Existing studies also revealed keywords or phrases related to specific MH conditions to be helpful. For example, in depression detection, researchers [54] identified prominent usage of words associated with depressive symptoms, such as “depressed”, “hopeless”, and “worthless”, referenced from the Depression Vocabulary Word List https://myvocabulary.com/word-list/depression-vocabulary/ (accessed on 10 December 2023), as well as antidepressant names [123] based on the list from Wikipedia https://en.wikipedia.org/wiki/List_of_antidepressants (accessed on 10 December 2023). Nevertheless, MH-related keywords may be expressed differently for various MH disorders. For instance, individuals with schizophrenia used more words related to perception (hear, see, feel), swearing, and anger [204], while Tébar et al. [217] found individuals with an eating disorder (ED) publishing less ED-related content, involving fewer indicative terms like “laxative names” and “weight concerns”, to keep their illness private. The latter study demonstrated false positives introduced by such disparities, as healthy controls were involved in discussions of MH disorders like PTSD or depression that share some ED symptoms or mentioned prevalent topics in the pro-ED community.

#### 3.3.4. Social Media

On top of user-generated texts and images on social media platforms, researchers could infer social networks and interactions from metadata associated with users and posts, where followers and followings could indicate “friendships”, whereas interactions like posting, liking, and commenting could reveal social interactions and topics of interest. While most platforms offer fundamental post information such as time posted, likes, and comments, some details are platform-specific, such as retweets (Twitter), check-in locations (Facebook), favorites (Twitter), profile images (Instagram), and users’ details like age and gender (Sina Microblog). Graph architectures could then be adopted to model the information above, for instance, by having a node for each user and an edge between two nodes representing the presence or extent of particular social interactions.

Research attempts have demonstrated a significant association between time spent on social media platforms [116] and depressive symptoms. This claim is supported by compelling evidence indicating that a substantial proportion of individuals with depression (76% [130]) and suicidal intent (73% [188]) engaged in more active posting activities on various social media platforms, including Instagram [125], Twitter [20,118], Reddit [130,188], and Weibo [189], particularly at midnight. Some authors [20,118,134] intuited this behavior as potentially linked to sleeping problems or insomnia. The corresponding posts by these individuals were also found to receive less engagement and attention, such as likes, retweets, and favorites [122,137]. Nevertheless, researchers observed contradicting trends in posting behaviors. While a few studies [122,125,137,189] revealed that those with MH disorders were generally less active on Twitter and Instagram, the opposite was observed in other studies on Twitter [121] and Sina Microblog [109]. Such disparities could be attributed to different user populations or sampling periods that may influence social behaviors on these platforms. Other potentially depressive behaviors include less disclosure of personal information [123], a greater likelihood of modifying images before posting [220], and lower preferences for sharing location [122].

Several research attempts emphasized the role of social networks in identifying MH disorders, where researchers incorporated public information belonging to other social media users engaged through followings, likes, comments, and tweet replies. For instance, Liaw et al. [134] and Ricard et al. [110] respectively involved liked content and that generated by users who have liked or commented on posts created by individuals of concern. The prior found the amount of depression keywords in liked content to contribute the most performance gain, whereas the latter found an improvement after incorporating such community-generated data. Similarly, Pirayesh et al. [138] and Mihov et al. [139] incorporated content created by homogeneous friends identified through clustering and computation and noticed improvement after increasing the number of homogeneous friends and their respective tweets.

#### 3.3.5. Smartphone and Wearable Sensors

Smartphone sensor data could be utilized to gain an understanding of individuals’ mobility (e.g., accelerometer, gyroscope, GPS data), sociability (e.g., call logs, text messaging logs, usage of social applications), and environmental context (e.g., ambient light and sound, wireless WiFi and Bluetooth connections). More personalized insights could be obtained by utilizing location semantics; grouping mobile applications into social, engagement, and entertainment categories; and detecting periodicity and routines to infer individuals’ behaviors and routines. Wearable devices further complement smartphone sensor data by offering sleep inferences and physiological signals like heart rate, skin temperature, and calories. Research attempts have uncovered potentially significant indicators of the presence or severity of MH disorders, which we explained in detail in the following paragraphs revolving around three primary aspects, i.e., physical mobility, phone interactions, and sociability.

(1) Physical Mobility Features: Studies have shown that negative MH states and greater depression severity are associated with lower levels of physical activity, demonstrated via fewer footsteps, less exercising [154], being stationary for a greater proportion of time [205], and less motion variability [149], whereas a study on the student population showed an opposite trend for increased physical activity [157]. Movements across locations in terms of distance, location variability, significant locations (deduced through location clusters) [177], and time spent in these places [164] were also valuable. For instance, researchers found greater depression severity or negative MH states associated with less distance variance, less normalized location entropy [154,158], lower number of significant visited places with increased average length of stay [158], and fewer visits to new places [205]. In contrast, Kim et al.’s [162] investigation on adolescents with major depressive disorders (MDD) found that they traveled longer distances than healthy controls. Timing and location semantics could further contribute more detailed insights, such as the discoveries of individuals with negative MH states staying stationary more in the morning but less in the evening [205], those with more severe depression spending more time at home [154,175], and schizophrenia patients visiting more places in the morning [206]. Researchers also acquired sleep information either through inferences from a combination of sensor information relating to physical movement, environment, and phone-locked states or through the APIs of sleep inferences in wearable devices. Sleep patterns and regularity were demonstrated to correlate with depressive symptoms [150,158] where individuals with positive MH states wake up earlier [205], whereas MDD patients showed more irregular sleep (inferred from sleep regularity index) [149].
(2) Phone Interaction Features: Phone usage (i.e., inferred from the frequency and duration of screen unlocks) and application usage were potentially helpful. For instance, several studies [158] found a high frequency of screen unlocks and low average unlock duration for each unlock as potential depressive symptoms. However, while Wang et al. [205] demonstrated the association between negative MH states and lower phone usage, the opposite trend was observed in students and adolescents with depressive symptoms who used smartphones longer [150,162,164]. Researchers also investigated more fine-grained features, such as phone usage at different times of the day, where they found schizophrenic patients exhibiting less phone usage at night but more in the afternoon [206]. Additionally, individuals with MH disorders also showed distinctive application engagement, such as Opoku Asare et al.’s [166] findings that individuals with depressive symptoms used social applications more frequently and for a longer duration. Generally, they also showed more active application engagement in the early hours or midnight compared to healthy controls, who showed diluted engagement patterns throughout the day. Meanwhile, Choudhary et al. [212] revealed that individuals with anxiety exhibited more frequent usage of applications from “passive information consumption apps”, “games”, and “health and fitness” categories.
(3) Sociability Features: Sociability features, such as the number of incoming/outgoing phone calls and text messages and the duration of phone calls, were also potential indicators of MH disorders [164,175]. For instance, negative MH states are associated with making more phone calls and text messaging [205,222] and reaching out to more new contacts [222]. On the other hand, adult and adolescent populations suffering from MDD were revealed to receive fewer incoming messages [149] and more phone calls [162], respectively. Lastly, ambient environments could also play a role since individuals with schizophrenia were found to be around louder acoustic environments with human voices [206], whereas those with negative MH states demonstrated a higher tendency to be around fewer conversations [205] than healthy controls.

#### 3.3.6. Demographics and Personalities

In addition, demographics and personalities might play a role in an individual’s responses to MH disorders. For instance, several studies [25,109] proved that females have a higher tendency to exhibit depressive symptoms than males. Individuals of different genders may also express varying responses to MH disorders to different extents. For instance, Yazdavar et al. [25] found that females expressed depressive symptoms more prominently on social media, implying their strong self-awareness and willingness to share their encounters to seek support. Meanwhile, a study [219] revealed that age, emotions, and the usage of words related to personal concerns are among the most significant indicators for identifying female samples with potential risks of anorexia nervosa, whereas words relating to biological processes were more indicative for male samples. Clinical experts involved in the study further identified gender as one of the most relevant factors to consider in locating anorexia nervosa. Fine-grained visual elements like formant, eye gaze, facial landmarks, and head pose may also vary across genders with depressive symptoms [70].

On the other hand, existing works [286,287] proved the potential association between MH symptoms and personality traits, where pursuing perfection, ruminant thinking and interpersonal sensitivity could be markers of suicide risk [287], whereas conscientiousness and neuroticism exhibited close relations to depression cues [121]. Researchers have estimated the personality scores of study samples based on textual content, for example, using IBM’s Personality Insights https://www.ibm.com/cloud/watson-natural-language-understanding (accessed on 10 December 2023) [57,121] or computing the proportion of words relevant to those in perfection- and ruminant-thinking-related lexicons [187]. Specifically, Chatterjee et al. [188] uncovered that 56% of suicidal samples demonstrated the association between low agreeableness and high neuroticism scores with increased suicide ideation, compared to most healthy controls with high agreeableness and optimism scores. Another study [130] also found that individuals with depressive symptoms generally have higher neuroticism and lower optimism scores.

### 3.4. Modality Fusion

#### 3.4.1. Feature Transformation to Prepare for Fusion

Some studies further applied transformation on extracted features to prepare for fusion by achieving (1) normalization, (2) dimensionality reduction, and (3) feature alignment. Normalization ensures that numerical features share similar scales and are treated equally by ML models. The most common normalization approaches that we observed are min-max normalization, to scale values between 0 and 1, and z-normalization [288], so that values are zero-mean and unit-variance. Since min-max normalization was claimed to preserve data relationships without reducing outlier effects [56], Cao et al. [187] took this inspiration to represent a subject’s age relative to the maximum age among all subjects in the dataset. Meanwhile, dimensionality reduction approaches were widely adopted, such as principal components analysis (PCA), singular value decomposition (SVD), and factor analysis. Lastly, for feature alignment, researchers transformed feature representations of individual modalities to align their dimensions through whitening (ZCA) transform (sparse coded feature representations) [49], global max pooling [77], or discrete Fourier transform (express visual features in the time–frequency domain) [66]. On the other hand, several other studies adopted neural networks to enforce the exact dimensions of multimodal representations. For example, using fully connected (FC) layers with the same units to condense features to a uniform dimension [70,112,179], a multilayer perceptron (MLP) [111], or bidirectional gated recurrent unit (Bi-GRU) [106] to transform representations to specific dimensions and a custom transformer-based architecture that applies linear projection to match various representation sizes [174]. In addition, an FC layer was also used to embed categorical variables to be concatenated with continuous variables [170].

#### 3.4.2. Multimodal Fusion Techniques

Multimodal fusion techniques combine features extracted from different modalities (e.g., audio + visual + textual data) into a single representation for training an ML model. Inspired by an existing work [69], we categorized existing fusion techniques into three main classes, i.e., at the feature, score/decision, and model levels. We hereby emphasize that the current discussion excludes scenarios where fusion is not required if modality-specific features are in independent numerical forms, which ML algorithms could be applied directly.

A feature-level fusion is also known as early fusion, where the features of all modalities are concatenated directly before feeding into an ML model. At the score/decision level, instead of features, researchers combined scores/decisions predicted by individual ML models for each modality, such as probabilities, confidence scores, classification labels, or other prediction outcomes, through operations such as AND, OR, product-rule, sum-rule, and majority voting. Fusing these scores would either produce the final outcome or serve as the input to a secondary ML model. There were also hierarchical score/decision-level fusion approaches that aggregate outputs across multiple layers or stages. For example, in Chiu et al.’s [122] user-level depression classification from social media data, the authors first obtained day-based predictions from post-level outputs weighted based on time intervals. Then, they deduced user-level outcomes based on whether day-based predictions fulfilled predefined criteria.

Unlike feature-level fusion, which concatenates features directly into a single representation, model-level fusion methods utilize an architecture or ML model to learn joint representations that consider the correlation and relationships between feature representations of all modalities. For instance, attention-based architectures (e.g., attention layers, transformers with multi-head attention mechanisms) were adopted to learn shared representations incorporating modality-specific representations with varying extents of contributions based on their significance. Meanwhile, cross-attention mechanisms were employed to consider cross-modality interactions. Shen et al. [20] also proposed using dictionary learning to learn multimodal joint sparse representations, by claiming that such representations are more effective than using features directly as the inputs of ML models. Nevertheless, we acknowledge the limitation that our categorization is merely based on our understanding, and specific fusion techniques in each category may implicitly involve a combination of various fusion levels. For a complete list of studies, methods, and tools, see Appendix B.

### 3.5. Machine Learning Models

Previous studies adopted ML models for binary classification on the presence of specific MH disorders, multi-class classification on the stages of MH disorders, and regression on the score based on an assessment scale. A complete overview of these models and their application methods is available in Appendix C. Referring to an existing study [289], we classified them into:

- Supervised learning—trained on labeled input–output pairs to learn patterns for mapping unseen inputs to outputs.
- Neural-network-based supervised learning—a subset of supervised learning algorithms that mimics the human brain by having layers of interconnecting neurons that perform high-level reasoning [290] to recognize underlying relationships in data [291].
- Ensemble learning—combines multiple base learners of any kind (e.g., linear, tree-based or NN models) to obtain better predictive performance, assuming that errors of a single base learner will be compensated by the others [292].
- Multi-task learning—attempts to solve multiple tasks simultaneously by taking advantage of the similarities between tasks [289].
- Others—incorporates semi-supervised, unsupervised, or combination of approaches from various categories.

#### 3.5.1. Supervised Learning

The availability of ground-truth information, obtained via expert annotations or clinical assessments, has enabled the broad application of supervised learning approaches that learn the association between input data and their labels. In our findings, these approaches primarily cater to univariate features, where feature engineering may be required to apply them to multidimensional data. The more popular ML algorithms for supervised learning are linear regression, logistic regression, and support vector machines (SVMs). Based on comparisons conducted in existing studies, stochastic gradient descent [43] and least absolute shrinkage and selection operator (lasso) regression [200,213] models performed the best in respective investigations on different feature combinations, i.e., the prior on audio, visual and textual features and the latter on wearable sensor signals, but these models are yet to be compared under similar settings. In addition to the traditional or linear algorithms mentioned above, the following subsection discusses a subset of supervised learning approaches utilizing neural networks.

#### 3.5.2. Neural-Network-Based Supervised Learning

A neural network (NN) [290] is fundamentally made up of an input layer, followed by one or more hidden layers, and an output layer. Each of these layers consists of neurons connected through links associated with weights. An FC layer is included in specific architectures to perform high-level reasoning since it connects all neurons in the previous layer to every neuron in the current layer to generate global semantic information [291]. Meanwhile, an architecture is considered a deep neural network (DNN) when more hidden layers are involved. Although NN architectures could be utilized for various learning approaches, such as supervised, semi-supervised, unsupervised, and reinforcement learning [293], this subsection only concerns those utilized for supervised learning tasks. In such contexts, an NN algorithm approximates a function that maps data received by input neurons to outputs via output neurons by adjusting weights between connected neurons [290]. Therefore, NNs can receive numerical data and yield outputs of any dimension, aligning with the corresponding number of neurons in the input and output layers, respectively.

Throughout this work, we have observed vast applications and versatilities of NN-based models in feature extraction, modality fusion, and ML prediction, which could be applied directly to multidimensional signals or transformed feature representations. As such, we raise the attention of future researchers to the potential overlapping between the NN-based approaches adopted in the three stages above. For example, the outputs of specific hidden layers in such models applied to raw signals or low-level features could be extracted as high-level feature representations, whereas those from the output layers could be utilized as prediction outcomes. The NN model in such scenarios could then be treated as either a feature extraction technique or an algorithm. The same applies to specific sophisticated architectures proposed to capture cross-modality interactions in model-level fusion, where these networks learn fused representations while simultaneously generating predictions.

The abundance incorporation of LSTM [294] for its capability of capturing temporal information across long sequences emphasized its potential. Transformer-based models [295], such as BERT [266] (including its variants like RoBERTa [296], ALBERT [297], EmoBERTa [298]) and XLNet [285], also gained popularity due to their capability to effectively capture contextual information through positional encodings [129] and attention mechanisms to learn different significance weights of relevant information. In contrast, some researchers incorporated attention mechanisms into existing NN architectures such as FC layers, LSTM, and GRU to achieve such emphasis. Despite demonstrating satisfactory efficacy, existing researchers obtained inconsistent findings regarding the influence of NN architecture complexity on the resulting effectiveness. For example, stacking NN architectures, like GRUs [119], CNNs [60], and LSTM [215], improved performance on top of utilizing baseline architectures such as those on both hand-crafted univariate features and raw signals. However, a few studies proved simple shallow NN-based models to succinctly outperform deeper architectures, for instance, AlexNet outperforming VGG-16 and RestNet101 [122], and a 2-layer Bi-LSTM which outperformed LSTM and GRU of varying layers [220].

Overall, the capabilities of NNs in learning high-dimensional data offer promising effectiveness and flexibility in mental healthcare involving heterogeneous data for capturing multifaceted aspects of MH disorders. Nevertheless, such models require large, high-quality datasets since they can only learn patterns within the training data [290]. Due to the complex and non-linear structure with multiple hidden layers, black-box NNs further introduce challenges in obtaining interpretable explanations of how the algorithms arrive at an output [16,299].

#### 3.5.3. Ensemble Learning

Ensemble learning algorithms have shown remarkable effectiveness by combining base models with similar or complementary learning principles [173]. Similar to supervised learning, such algorithms were applied to univariate inputs, which could be hand-crafted numerical features or predicted outputs (e.g., regression scores, probabilities, binary labels) from other baseline models. The few popular ensemble learning approaches are tree-based, such as random forest (RF) [300], eXtreme Gradient Boosting (XGBoost), AdaBoost [301], and Gradient Boosted Regression Tree [302], which utilize decision trees as fundamental. XGBoost and AdaBoost were gradually favored by researchers due to their better predictive performance. Specifically, few studies [134,158,184,212] revealed XGBoost as the most effective among SVM, RF, K-nearest neighbor, logistic regression, and DNN models. In contrast, researchers also proposed novel hierarchical ensemble architectures by stacking algorithms (e.g., XGBoost [194], Extreme Learning Machine (ELM) [192]) into layers where models in subsequent layers receive outputs from previous layers as inputs for ensemble predictions. For example, Mishra et al. [185] and Liu et al. [123] adapted the feature-stacking [303] approach by utilizing logistic regression to combine predictions of various first-level learners, like SVM, KNN, and Lasso regression, applied independently to different feature sets. In addition, Tabassum et al. [168] combined an LSTM-based model applied to hourly time series sensor data and an RF on statistical features aggregated across the data collection duration to benefit from the strengths of respective learning algorithms.

#### 3.5.4. Multi-Task Learning (MTL)

Unlike ensemble learning, MTL involves a single model (of any category mentioned above) trained to solve several simultaneous tasks to exploit task-specific similarities and differences. Examples of task combinations are (1) regression and classification [74,102,118,141,193], (2) depression prediction and emotion recognition [46,106,132], and (3) gender-specific predictions [70]. Though most of the included studies adopted NN-based models, such as CNN [61,62], LSTM [46,106], and DNN architectures [102,193], MTL could also be achieved with linear models, for example, the multi-output support least-squares vector regression (m-SVR) [304] trained to map multivariate inputs to multivariate outputs [207]. Meanwhile, Oureshi et al.’s [70] findings further justified the role of demographics in locating MH disorders, such that incorporating gender prediction as an auxiliary task improved the overall performance.

#### 3.5.5. Others

We observed a few studies applying unsupervised techniques, with clustering using K-nearest neighbors being the most common approach. A few other researchers also adopted anomaly detection using existing unsupervised techniques like Isolation Forest (ISOFOR) [166], or statistical measures such as *t*-tests for detecting outliers among preliminary prediction outcomes [163]. The research attempts mentioned above revealed that these unsupervised approaches appear more promising on smartphone sensor data than conventional ML approaches, including SVM, RF, GDBT, and MLP. In addition, AbaeiKoupaei et al.’s work [196] was the only semi-supervised learning we identified in this study, in which the authors employed a ladder network classifier [305] consisting of stacked noisy encoder and denoising autoencoder [306]. There were also novel approaches adapting various concepts, including recommender system (RS) [173,307], node classification [173], and federated learning [168]. Additionally, some studies employed computations to learn association parameters [83,189] or deduce prediction outcomes from distance-based homogeneity [85].

### 3.6. Additional Findings

#### 3.6.1. Modality and Feature Comparisons

Most studies on multimodal detection justified the effectiveness of combining multiple modalities due to their complementary outcomes, which outperformed unimodal approaches. Notably, we noticed a single exception in a finding [174] that textual modality alone is succinctly effective, such that combining it with audio and visual modalities slightly deteriorated the overall performance. Deeper analyses also revealed that specific modalities could be more influential than others. From audio-visual recordings, semantic content in audio transcriptions generated via textual features was found to be more indicative of depression than audio and visual features in several studies on depression, bipolar disorder, and suicidal ideation. Specifically, we found such prominence arising from textual representations using various embedding techniques like GloVe [49], Universal Sentence Encoder [59], Paragraph Vector [102], and ELMo [116].

On the contrary, several revelations highlighted the great potential of audio MFCC features. For example, a study [65] attempting to detect depression in audio samples of less than 10 seconds, another [72] conducted on Chinese language audio samples, and one on detecting bipolar disorders [103] found MFCC features more effective than textual embeddings. Nevertheless, more fine-grained comparisons are required to justify the efficacy of one modality or modality-specific feature over the other due to the varying influence of experimental contexts and setups in data collection and feature extraction.

#### 3.6.2. Personalized Machine Learning Models

In conjunction with an existing finding that individuals with similar depression scores may portray behavioral differences under similar contexts [156], several researchers attempted to achieve individual personalization by training subject-specific models [164,169,205,207], fine-tuning subject-specific layers [161] in a global NN architecture, and deducing personalized predictions by incorporating information from other samples homogeneous to each individual based on correlation coefficients [156] or demographics [208] such as age [209].

Meanwhile, existing attempts at gender-based subgroup personalization also highlighted the potential significance of gender in identifying MH disorders. Researchers achieved such personalizations via training the same ML models on gender-specific samples [92,94,219], fine-tuning and building individual ML models for each gender subgroup [48,161], or incorporating gender prediction as an auxiliary task in an MTL approach [70]. Nevertheless, existing researchers found contradicting findings of models constructed from gender-specific samples. For instance, Pampouchidou et al. [48] and Samareh et al. [54] proved that gender-based classification models outperformed gender-independent ones, whereas others [92,94] demonstrated that global models trained on all genders predicted gender-specific evaluation instances more effectively than those trained on gender-specific data. Attempts above [92,219] further uncovered challenges in effectively predicting female samples, where the outcomes indicated that gender-specific models trained and evaluated on female samples perform worse than those of male samples.

## 4. Discussion

### 4.1. Principal Findings

This section addresses the research questions based on findings in the previous section.

#### 4.1.1. RQ1—Which Sources of Data Are Most Effective for Supporting the Detection of MH Disorders?

**Figure 5:**
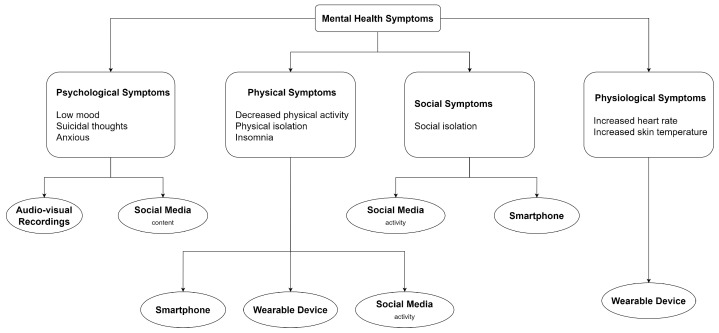
Mapping of data source to mental health symptoms.

Our findings have explored evidence of associations between specific modality-specific features and various MH symptoms. In Figure 5, we categorized MH symptoms into psychological, physical, social, and physiological aspects [19] and mapped these aspects to the capabilities of data sources in capturing them. The figure illustrated that no multimodal data source can capture all aspects of MH symptoms and that deducing the most effective data source relies on the symptoms researchers wish to investigate that are relevant to specific MH disorders (see Table 4 for existing studies utilizing specific data source to investigate each MH disorder). We acknowledge that the figure only includes mapping relevant to passive data sources included in the current study and that other active sensing approaches may be valuable and complementary.

Psychological symptoms related to moods, emotions, and feelings were shown to be effectively captured by textual features, which could be obtained from transcriptions of audio-visual recordings and the content of social media posts. For example, individuals with MH disorders expressed stronger negative emotions via texts with overall negative sentiment or more negative words and emoticons, as well as through MH-specific keywords related to symptoms, treatment, and medications (e.g., antidepressant names and phrases associated with depressive symptoms for depression). While these textual features have been proven indicative, researchers found visual cues to provide complementary information by encapsulating finer details of individuals’ implicit inner emotions. For example, the significant association between FAUs and depressive symptoms may indicate how individuals present their facial expressions in response to MH symptoms. In addition, individuals’ publicly shared images may reflect their psychological conditions; for instance, preferences for darker colors may represent lower moods, or images of animals may represent self-coping mechanisms to improve emotional states.

Meanwhile, for physical symptoms, the unobtrusiveness and ubiquity of smartphones and wearable devices have great potential to capture individuals’ natural behaviors, which could reflect the physical manifestations of MH-specific symptoms. For example, the association of higher depression severity with lower physical mobility, demonstrated via being stationary for a greater proportion of time or traveling to fewer places sensed using GPS and accelerometer, may suggest depressive symptoms of losing interest in surroundings, lethargy, or social isolation. Wearable sensors could complement by offering sleep-related information like sleep states and duration to infer sleep quality. In addition, individuals’ social media activities could reflect their personal routines and behaviors, for example, higher susceptibility to insomnia or sleeping problems implied through frequent posting activities during midnight. In contrast, a decline in social interactions is an example of social symptoms that might indicate a reduction of interest in surroundings. Such social interactions could include both verbal communications detected via microphones in smartphones and social interactions made through social media platforms and social mobile applications. Lastly, wearable devices are the only passive data source capable of tracking changes in physiological symptoms, including heart rate, skin temperature, and calories burnt.

Nevertheless, our findings in Section 3.3.6 highlighted the influence of demographics and personalities on individuals’ behaviors, where specific subgroups may openly share their symptoms to seek external support [25], while some may show reluctance through prominent usage of the word “others” or third-person pronouns on social media [123,187]. Additionally, Shen et al. [109] proved the divergence in Twitter and Sina Microblog user behaviors, where those with depressive behaviors posted less frequently on Twitter, but the opposite trend was observed in Sina Microblog users. There was also a higher occurrence of positive words in textual content in the latter than in the prior. Such disparity in expressivity could be attributed to different populations’ cultural and language differences since Sina Microblog users are primarily from Asian countries. Meanwhile, an increased regularity in physical activities could be a coping mechanism for young adults [157] but is not necessarily the case for other contexts or populations having a depressive symptom of lacking interest in activities, as shown in several other studies [110,149,150,152,158].

In addition to modality-specific effectiveness, discussed in Section 3.3, there is a need for deeper considerations beyond data source, which are associated with the experimental contexts of data collection approaches and the individuals to which the data belong. We established several criteria to evaluate data collection approaches in greater detail in Section 4.2 below.

#### 4.1.2. RQ2—Which Data Fusion Approaches Are Most Effective for Combining Data Features of Varying Modalities to Prepare for Training ML Models to Detect MH Disorders?

Based on our categorization of modality fusion techniques, namely feature, score/decision, and model levels, we recommend employing feature or model-level fusion based on researchers’ specific use cases. Our observations suggest that score-level fusion might be less effective, as modality features are modeled separately by individual ML algorithms, thereby ignoring the potential correlation of features across different modalities. As previously discussed, certain modalities may be more effective than others in unimodal settings. Since score-level fusion only considers the intermediate prediction outcomes of modality-specific ML models, the more effective modalities may overshadow the less significant ones in the final outcomes, even though all modalities are complementary. The following paragraphs provide more detailed recommendations to decide between feature and model-level fusion.

Feature-level fusion is readily applicable to simple univariate features, where direct concatenation is straightforward and efficient to implement. Researchers should align their decision with research objectives by considering whether the features succinctly capture the information they intend to investigate with sufficient details and the capability of adopted ML algorithms to model the correlation among such features to answer specific research questions. For example, a high-level aggregation by computing the average steps, distance traveled, and time spent at an individual’s home across the entire study duration produces a simple univariate vector representation for feature-level fusion, but such information may not be relevant for a study intending to capture daily variations in individuals’ physical behaviors. If researchers intend to prioritize efficiency and low computational complexity, specific computation methods should be investigated to effectively elicit low-dimensional representations that capture time-based variations. For instance, autocorrelation analysis captures feature periodicities across specific durations [157] where the resulting correlation coefficients could be utilized as higher-level representations of time series data.

On the other hand, model-level fusion has been shown in several attempts [69,71,73,308] to generate more effective high-level feature representations than hand-crafted univariate features due to their capability of capturing temporal and contextual dependencies while modeling cross-modality interactions. The decisions of whether to adopt model-level fusion and the architecture to employ should consider the complexity of the research problems. For example, deep NN architectures with more extensive layers may be more effective if researchers are interested in investigating the influence of particular factors across long durations. However, researchers should be aware of the greater computational costs associated with more complex architectures and the black-box nature of certain NN-based architectures, which reduce the interpretability of modeled interactions.

We have observed an increased adoption of various NN architectures among researchers to model feature information at varying complexity levels. Several architectures have shown outstanding efficacy by incorporating both temporal and contextual interactions within and across modalities, underscoring the importance of generating fused representations that encapsulate such information. For instance, Yan et al. [170] applied Convolutional Sequence Embedding Recommendation (CASER) [309], which leverages convolutional filters of CNNs. In CASER’s horizontal convolutional layer, the authors applied convolutional filters horizontally to capture daily-level sequential patterns as local features for all feature points at the previous time step, followed by max-pooling to extract the most meaningful information. In the vertical convolutional layer, feature-level patterns were generated as the weighted sum of each feature point at specific time steps, with the convolutional filter acting as weights. The outputs of both convolutional layers were then concatenated into fully-connected layers to produce fused representations. This architecture demonstrated more effective capture of hidden series patterns than aggregated statistical features (e.g., average, minimum, or maximum across a duration). In contrast, Zhou et al. [93] proposed a time-aware attention multimodal fusion (TAMF) network. This architecture includes a sparse MLP, utilizing its weight sharing and sparse connections to mix information from modality-specific representations in both vertical and horizontal directions. The resulting outcome is a mixed attention vector, which is separated into attention vectors of each modality. The final fused representations were obtained by summing modality-specific representations weighted by respective attention vectors. TAMF was claimed to model the importance of different modalities at different times, with well-rounded consideration of cross-modality interactions.

#### 4.1.3. RQ3—What ML Approaches Have Previous Researchers Used to Successfully Detect MH Disorders from Multimodal Data?

We could not deduce a single one-size-fits-all model that is the most effective for various multimodal tasks. This is because the effectiveness of ML algorithms relies on the nature and structure of the data, the task to achieve, and how data information is learned and fully utilized. Nonetheless, our observations revealed that ML models adopted in existing studies are primarily supervised learning algorithms, which utilize ground truth as the “gold standard”, and several models worth investigating are Lasso regression, XGBoost, LSTM-based models, and transformer-based models like XLNet, based on their prominent predictive performance in existing studies. Notably, we noticed a similar trend of rising adoption of NN-based models in recent studies, which appear more valuable than linear statistical algorithms in modeling multidimensional time series signals. As previously discussed in Section 3.5.2, existing researchers have proposed various novel architectures to incorporate temporal, contextual, and cross-modality dependencies, such as injecting time-based representations into transformer models to improve performance further [129]. We also observed the significance of utilizing relevant data information, where a few studies [111,186] demonstrated that irrelevant textual and visual content introduced noise that obstructs traces of MH disorders and caused further performance deterioration. While the studies above selected more relevant information through techniques like reinforcement learning [111], other studies utilized attention mechanisms to exert significance weights based on relevance instead of eradicating less relevant information. Specifically, attempts [128,146] at hierarchical attention asserted onto deep representations of social media content from word level and subsequently to post and user levels highlighted the great prospect of such mechanisms.

Despite both audio-visual and sensor data being time series data, we noticed relatively limited applications of NN architectures on sensor data. Most studies employed conventional linear or statistical ML algorithms (e.g., logistic regression, SVM, XGBoost), which learn from univariate inputs, by aggregating extracted features across the whole duration. Such approaches potentially neglected the associations of features across time since several recent studies [162,170,176] proved the superiority of NN-based models applied to higher-dimensional time series sensor data, where features are aggregated at hourly, daily, or weekly features, over conventional univariate approaches. These outcomes suggested an aspect worth investigating for future researchers to better harness the potential of ML algorithms, for example, by applying an LSTM-based model to hourly time series data and RF to univariate features derived from the prior data to leverage the strengths of both algorithms [208].

While most studies predominantly focused on optimizing performance metrics like accuracy, precision, and recall, they often overlooked practical considerations for real-world applications of ML models, such as complexity, explainability, and generalizability. Despite the inherent biases in ML algorithms [310], only two included studies examined potential biases at the individual and gender levels. For ML models to be seamlessly integrated into real-time detection applications for clinical use, they must be lightweight in terms of complexity and computational cost, considering that available memory and computation resources may be restricted [311], especially in individuals’ local devices. Specifically, in the MH domain, this criterion is significant to enhance efficient computations on the fly and timely delivery of personalized interventions and recommendations without imposing excessive processing power [311]. Ultimately, achieving this can potentially improve individuals’ accessibility to mental healthcare resources and subsequently promote their treatment-seeking.

In addition, with the growing attention to the interpretability and explainability of ML models [299], these criteria offer transparency to ML models’ decision-making to establish trust in these algorithms and elevate their practicality in real-life applications. Specifically, in high-stake MH applications where black-box predictions potentially bring harmful consequences, explanations of ML outputs can provide meaningful insights for healthcare professionals to understand and validate the relevance of ML outputs in complementing clinical diagnosis. Considering the inherent biases in ML algorithms [310], transparency in working mechanisms (local explainability), feature contributions (global explainability [299]), and potential shortcomings are necessary for healthcare professionals to guide and manage the influence of ML outputs on clinical decisions.

Meanwhile, generalizability improves the transferability of ML models to external scenarios beyond local training environments. Given the potential influence of demographics and personalities on manifestations of MH-related behaviors, existing surveys [17] have highlighted the risks of under-representation of certain groups in training datasets, in which the demographic disparities may be magnified in the subsequent applications to the MH domain. Generalizability can enhance the applicability of ML models to other contexts and heterogeneous populations, subsequently improving the accessibility of the general population to MH resources. As an existing work [312] demonstrated the difficulties of aligning cross-study settings for improved generalizability, Thieme et al. [17] emphasized avoiding overclaiming premature generalization from datasets lacking clinical validation and diversity. As such, future researchers should validate and communicate potential limitations in the generalizability of research outcomes. For example, researchers should account for the diversity within a population by validating outcomes across different subgroups with various demographics and characteristics and reporting on the metadata of the community from which the data is collected.

### 4.2. Evaluation of Data Sources

Following the address of RQ1 in Section 4.1.1 above, we established several criteria to further analyze the different categories of data sources.

#### 4.2.1. Criterion 1—Reliability of Data

The reliability of a data source relies on how well it captures people’s real-life behaviors. This criterion is crucial to contribute relevant data for supporting clinical diagnosis since failure to reflect realistic behaviors may result in misdiagnosis of MH disorders, potentially leading to severe complications.

We perceived that smartphone and wearable sensor data are the most reliable due to their ubiquity and a lower possibility of people “tricking” sensors into gathering perceivably desired data. Prior to data collection, participants will configure dedicated mobile applications in their smartphones, allow permission to access specific sensor data, and establish wireless connections for wearable devices to their smartphones, where applicable. Afterwards, they will interact with their devices as usual throughout the data collection process with minimal active inputs. Given that they are open to and allow monitoring over a longer period, the awareness of monitoring may be reduced following the initial novelty effect, thereby enhancing the “honesty” of corresponding data. Nevertheless, researchers should consider data quality that can be affected by sensors of different devices with varying sensitivity. The fit of wearable devices may also affect the accuracy and amount of data collected. For example, improper wearing or wearables slipping off [313] during sleep may end up collecting poor, noisy data. In addition, participants may forget to reapply sensors (e.g., following a shower) or feel discomfort from wearing it on their wrists or other parts of their body.

Both audio-visual recordings and social media data potentially suffer from biases introduced by individuals’ self-presentation concerns. For example, a person may behave differently under the pressure of continuous supervision, also known as the Hawthorne effect [314], to look generally appealing or hide any indicative behaviors potentially due to fear of judgment. Similarly, social media users might curate their public posts to appear presentable due to factors like the consciousness of unfavorable public perception or social stigma. In addition, a study [187] found that users express their thoughts differently in the hidden tree hole posts than in usual Sina Microblog posts. A tree hole is a microblog space whose author has committed suicide, and other users tend to comment under the last post of the passed one about their inner feelings and thoughts. Such posts were revealed to contain more self-concern and suicide-related words, thereby challenging the detection of MH through regular or public posts.

#### 4.2.2. Criterion 2—Validity of Ground Truth Acquisition

A well-justified ground truth is vital to represent people’s actual MH states. From a responsible innovation perspective, unrealistic ground truth can cause under or over-estimations in MH detection, which may escalate to introduce dangers, especially in disorders with crisis points, such as suicidal ideation or an eating disorder.

Clinical assessments are the only method yielding representative ground truth thus far because self-reports, whether in the form of responses in assessment scales or self-declaration in social media posts, are subject to self-presentation and recall biases. However, we noticed a possibility of verifying ground truth from self-reports by utilizing behaviors detected via smartphone and wearable sensing. These approaches typically request individuals to answer clinically validated assessment scales based on guidelines like the Diagnostic And Statistical Manual Of Mental Disorders, Fifth Edition (DSM-V) [315] to serve as baseline ground truth. The response to each assessment question corresponds to recalled behaviors over a specific duration. Taking Wang et al.’s work [150] as inspiration, behaviors inferred from sensor data can be mapped to individual DSM-V symptoms to verify ground-truth labels. Conversely, some researchers primarily rely on social media users’ self-identification of MH disorder diagnosis to acquire the ground truth of their MH states, usually through identifying keywords associated with specific MH disorders. Such acquisition risks under-identification since it depends on whether people took the initiative and felt comfortable sharing the information. Solans Noguero et al. [219] further proved that suicide-related lexicons were less comprehensive due to the likelihood of omitting explicit vocabulary and failing to identify implicit hints.

A reliable ground truth should always be supported by clinical validation, such as through a diagnosis by trained practitioners, reference of clinical evidence, or having clinical experts verify manual annotations, since relevant clinical knowledge is necessary to ensure the validity of ground-truth labels. Specifically, in the use case of social media data, where individuals’ data were crawled directly from these platforms, there are both practical and ethical considerations that need to be addressed when claiming a ground truth has been established. While time-consuming, future studies could consider ways to directly approach social media users where possible to verify their MH states and to actively gain their consent for their data to be used (or ensure that users are aware of the research aims at the very least).

#### 4.2.3. Criterion 3—Cost

We inspected costs in terms of (1) data accessibility, (2) external costs incurred for dedicated data collection equipment and tools, (3) processing power for transforming and analyzing data, and (4) storage space. These considerations are crucial in evaluating the practicality of research outcomes in real-life applications so that a cost-effective method can be easily deployed to benefit the target population.

We deduced that social media data are the cheapest to acquire from all aspects above. It is the most accessible since researchers can crawl public data online without accessing users individually or getting hold of their private information, given that they comply with the platforms’ terms and conditions (whether this is deemed ethical is another question). Relatively small processing power and storage space are required since crawled data is in the form of data entries. Additionally, features can be extracted from textual and visual content using processing tools available, such as LIWC [278], NLTK [280], and SEANCE [282] for texts and OpenFace [269], OpenCV [270], and OpenPose [271] for images. Audio-visual recordings are the most costly because they encapsulate rich data information that requires large storage space and extensive computation power to process audio and visual elements. In addition, this approach requires video cameras and microphones, which might have to be purchased beforehand, and consumes more effort in setting up the equipment at one or more locations based on device reception and coverage.

Since most populations generally own smartphones [316], the potential equipment cost for smartphone sensing is lowered. However, a substantial cost might be incurred if researchers are to provide smartphones to study participants without smartphones or to ensure consistency. In contrast, wearable devices are cheaper but less ubiquitous than smartphones since some participants do not see the necessity of possessing wearable devices (e.g., fitness watch, smartwatch) and consider them a luxury item. Though both approaches involve time series sensor signals, which may be high dimensional, the storage cost is still relatively economical compared to multidimensional video files. Nevertheless, we observed a novel application of federated learning in Tabassum et al.’s [168] work, which is potentially feasible for resolving storage and privacy concerns. The authors processed collected data and extracted features locally in individuals’ devices to obtain local features and parameters. These were then utilized to fine-tune local individual-specific ML models, which share and exchange higher-level parameters with a global-centric model. This approach significantly lowered transmission cost and storage space since server transmission is reduced from complex multidimensional data to numerical features and parameters while minimizing the risks of privacy leaks during transmission since raw data was discarded after local processing. As such, researchers should consider the factors discussed in Section 4.1.3 to ensure that ML models residing in local devices are highly deployable, such as being efficient and lightweight, to avoid consuming excessive local processing power.

#### 4.2.4. Criterion 4—General Acceptance

The general acceptability of people towards specific data collection approaches has the most direct influence on research involving human data. This criterion can be attributed to people’s openness and comfortability in allowing their data to be collected, which are often supported by their perceptions and concerns about the methods. The control they have over the sharing of their data may also be a contributing factor.

We inferred wearable sensing as the most acceptable because it gathers the least identifiable data (e.g., physiological signals like heart rate and skin temperature, activity levels, and sleep patterns) that is most unlikely to disclose people’s personal information. On the other hand, acceptability towards smartphone sensing is debatable. A study [100] discovered GPS to be the most acceptable compared to calendars, call logs, text logs, and contacts, and only one-third of study participants shared their smartphone logs. However, this is not necessarily the case for some with safety concerns about revealing their locations (e.g., not wanting to disclose their homes or concerns over being stalked [317]), and allowing access to call and text logs can also be perceived as privacy-invasive. In both smartphone and wearable sensing contexts, participants may or may not have control over the kinds of data collected from them, i.e., which sensors are enabled, depending on the approach design and configuration by researchers.

The acceptance of social media users for researchers to utilize their data for research purposes is also controversial. Researchers have presumed that social media users are open to and permit others to access their data since they opted to make it public in the first place [318]. Even though users have complete control of their public content, they are unaware and may oppose their data being accessed and analyzed for research without consent. Meanwhile, we hypothesize that audio-visual recordings are the least acceptable because it is highly invasive, and not all individuals are comfortable having their footage taken and monitored continuously. Even though existing research [313] found a general acceptance of being recorded using privacy-preserving video cameras that only capture participants’ silhouettes, such cameras may not apply to the current context that requires identifiable elements, like facial expressions, body gestures, and movements. Researchers have complete control of the data collection process, and there were contradictory opinions from participants themselves on whether they should have control over when and what is being recorded, e.g., by allowing them to pause at specific critical times [313].

As much as the ability to manage data sharing based on personal comfort can improve the acceptability of data collection, researchers should be aware of the resulting risks of biases and sparsity in data. A reduction in the unobtrusiveness of passive sensing and an increased likelihood of skewness will occur if participants constantly manage their data sharing. There will also be data sparsity issues if participants can selectively activate/deactivate specific sensors at random times. There are other means of establishing people’s trust in researchers to raise their confidence that their shared data will be kept secure and handled cautiously with safety procedures. For example, this can be achieved by offering transparency of what is being collected, why, and how they will be stored and handled.

#### 4.2.5. Overall Findings

Overall, smartphone sensing emerged as the most promising avenue. Our findings demonstrate abundant significant correlations between sensor features and MH symptoms, offering the potential to translate such connections into an individual’s physical manifestations in response to specific MH disorders. While symptoms associated with specific MH disorders may manifest differently, the capability of smartphone sensors to capture natural behaviors and variations across time provides a strong advantage.

However, the integration of smartphone sensing into MH applications demands further research due to several critical considerations that are yet to be addressed. Ethical concerns arise regarding whether it is privacy-infringing for researchers to access individuals’ private or personal behaviors, which they may be unwilling to disclose. Consequently, they may deliberately hide or alter their behaviors to “trick” the data collection system or withdraw due to privacy concerns. These issues contribute to the potential unreliability and sparsity of the resulting data, introducing challenges for technical researchers to seek solutions for data-driven ML algorithms, especially in supervised learning with a heavy reliance on ground truth labels. Despite the rich information in time series sensor-based data, there is still room for research to investigate techniques that fully harness its potential. While existing studies have demonstrated the efficacy of neural network architectures in modeling such high-dimensional data, the low explainability and high complexity of such architectures remain a critical challenge. Additionally, existing elicitation techniques are often informed by standard guidelines like DSM-V, potentially disregarding behaviors yet to be discovered. As such, it is imperative to establish a common ground between researchers and clinical experts that enables collaboration to investigate and interpret ML outputs to ensure clinically relevant outcomes.

We hereby acknowledge that the above represents our perspectives based on the current understanding and analysis and that it is essential for future researchers to critically evaluate and adapt the insights based on the evolving landscape of technology and methodological approaches to their specific use cases in the MH domain. We outline some guidelines in the following subsection to assist future researchers in making informed decisions.

### 4.3. Guidelines for Data Source Selection

In light of the various influencing factors of MH conditions and the necessary considerations for high-stakes applications involving vulnerable individuals, we have devised guidelines that future researchers can use in conjunction with Figure 5 above for selecting an optimal data source or combinations of data sources based on specific use cases.

1. *Define research objectives and scope:* Clearly defined research objectives and questions can guide researchers to determine the kind of information required to achieve the research goals and, subsequently, to evaluate the extent of the data source in accurately representing or capturing relevant information. Determining the scope of the study is crucial to pinpoint and assess the relevance of data information to ensure that collected data effectively contributes to the desired outcomes.
2. *Determine the target population:* Identifying the target population and its characteristics involves various aspects, including the targeted MH disorders, demographics, cultural backgrounds, and geographical distribution. These aspects are mutually influential since individuals’ behaviors and data may vary based on reactions to different MH disorders, with further influence caused by cultural backgrounds and demographics, such as age, gender, and occupation. Additionally, geographical distribution and economic backgrounds may influence an individual’s accessibility to a specific data collection tool. This consideration ensures that the data collected is representative and applicable to the population of interest, enhancing the overall effectiveness of the approach.
3. *Identify candidate data sources and evaluate their feasibility:* Evaluating the feasibility of each data source in light of the research objectives and target population identified above assists researchers in making informed decisions. Given the contexts and environments in which the target population is situated, researchers can assess which data source is the most practical and relevant. For example, researchers may consider employing remote sensing to introduce the unobtrusiveness of data collection for high-risk MH disorders or overcome geographical challenges. This assessment should consider its feasibility in terms of cost and accessibility, and it should be informed by Figure 5 to ensure that the selected data source can effectively capture relevant MH symptoms.
4. *Consult stakeholders:* Engaging stakeholders, including healthcare professionals, patients, and families, provides various perspectives of parties involved in supporting individuals with MH disorders. These consultations verify and offer insights into the acceptability and feasibility of data sources and help ensure that researchers’ decisions align with ethical considerations and stakeholders’ comfort.
5. *Ethical considerations and guidelines:* Researchers should further consult institutional review boards and established guidelines to ensure the compliance of data collection procedures with ethical standards and research practices. This step is crucial to safeguard participants’ rights and privacy, enhancing the credibility of the study.
6. *Assess the significance of ground truth information:* Evaluating the significance of ground truth information informs how researchers gauge its impact on the study and whether specific workarounds are necessary to enhance ground truth reliability and validity during data collection. This evaluation will then aid researchers in designing the data collection procedure and determining the extent of reliance on ground truth to support future analysis, reasoning, and deductions.

## 5. Conclusions

This study examines existing methodologies for non-intrusive multimodal detection of MH disorders and critically evaluates various data sources in terms of reliability, ground truth validity, cost, and general acceptance. Given the complexity of identifying the most effective data source for detecting MH disorders, our guidelines offer a systematic approach for future researchers to make informed decisions about a data source that aligns with research objectives, is relevant to the target population, and adheres to ethical standards. In addition, our analysis highlights the potential of neural network architecture in model-level fusion for capturing higher-complexity cross-modality interactions. We also observe the prospect of utilizing such architectures as ML algorithms to handle high-dimensional data, though practical aspects, such as complexity, explainability, and generalizability, should be scrutinized beyond effectiveness.

We acknowledge the inherent limitations in our approach, recognizing that our search strategy might have omitted potential data sources not explicitly defined within our predetermined categories. Though our findings verified the significance of multimodality compared to unimodality in most cases, there is no absolute answer since the overall efficacy depends on modality-specific features. In addition, there are risks associated with our assumption that passive sensing captures natural behaviors and is more acceptable. The deliberate exclusion of active sensing based on this assumption limits our understanding of potential insights that active sensing approaches can offer. In conjunction with our previous discussion on seeking validation in ground truth information, active inputs may be valuable and necessary to achieve robust validation. As we critically evaluated each data source, we observed a refutation of our assumption, such that passive sensing approaches can be privacy-invasive and are not necessarily well accepted. This is due to the uncertainties and unobtrusiveness of such approaches, which may introduce a sense of insecurity among individuals from whom the data is collected. Building upon the acknowledgements, the current study has recognized smartphone sensing as a promising avenue for further exploration as our next step forward. In light of the ethical considerations and limitations identified, we plan to conduct interviews and focus groups with individuals with MH disorders to gather feedback on the acceptability of smartphone sensing and potential workarounds for addressing privacy concerns. Simultaneously, consulting healthcare professionals will provide valuable perspectives on incorporating smartphone sensing into clinical practice. As we embark on the journey into smartphone sensing, we extend an open invitation for collaboration with fellow researchers, healthcare professionals, and stakeholders passionate about advancing in this domain.

Nevertheless, our work aspires to bring significant implications for stakeholders, including researchers, mental healthcare professionals, and individuals with MH disorders. Our overview of current methodologies for handling multimodal data serves as a starting point for future MH researchers to explore methodological advancements for more effective and timely detection approaches. Our guidelines for data source selection provide a systematic approach for researchers to make informed decisions aligned with use cases or specific symptoms of interest. In addition, our critical analysis of passive multimodal data sources and modality-specific features provides insights to explore the effectiveness of other modality combinations for specific MH disorders. Subsequently, this inspires the development of specific tools that leverage external or multiple data sources to support mental healthcare professionals in their clinical practice (e.g., drawing inspiration from the beHEALTHIER platform [319] which integrates different types of healthcare data, including health, social care, and clinical signs, to construct effective health policies). We envision engaging with MH professionals through workshops, webinars, or other collaborative efforts to bridge the gap between research and practice. Additionally, our practical insights emphasize implementing ML approaches in real-world settings, paving the way for practical implementations that enhance the accessibility for individuals with MH disorders. The outcomes related to the correlation between specific inferred behaviors and MH symptoms also contribute to a better understanding of MH symptoms. Moving forward, we anticipate close collaboration with mental healthcare professionals and individuals with specific MH disorders to design a multimodal approach that facilitates more effective detection. Regardless, we acknowledge the need to establish a middle ground to effectively communicate technical concepts and implications to both stakeholder groups.

## Figures and Tables

**Table 1:** Search categories and keywords.

Category	Keywords
Mental disorder	Mental health, mental disorder, mental illness, mental wellness, mental wellbeing
Method	Artificial intelligence, machine learning, model
Outcome	Detect, predict, classify, monitor, recognize, identify
Data source/modality	Social media, text, speech, voice, audio, visual, image, video, smartphone, mobile, wearable, sensor

**Table 2:** Data to extract to answer respective research questions.

ID	Item	RQ
I1	Reference (authors and year)	N/A
I2	Title	N/A
I3	Mental health disorder investigated	N/A
I4	Data collection process	RQ1
I5	Ground truth/data labeling	RQ1
I6	Feature extraction process	RQ2
I7	Feature transformation process if any	RQ2
I8	Feature fusion process	RQ2
I9	Machine learning model	RQ3
I10	Results achieved	N/A
I11	Analysis findings if any	N/A

**Table 3:** Quality assessment criteria and scoring.

ID	Criteria	Scoring
QC1	Was there an adequate description of the context in which the research was carried out?	The design, setup, and experimental procedure are adequately (1), partially (0.5), or poorly described (0)
QC2	Were the participants representative of the population to which the results will generalize?	The participants fully (1), partially (0.5), or do not (0) represent the stated target population
QC3	Was there a control group for comparison?	Control group has (1) or has not (0) been included
QC4	Were the measures used in the research relevant for answering the research questions?	Adopted methodology and evaluation methods are fully (1), partially (0.5), or not (0) aligned with research objectives
QC5	Were the data collection methods adequately described?	Data collection methods are adequately (1), partially (0.5), or poorly (0) described
QC6	Were the data types (continuous, ordinal, categorical) and/or structures (dimensions) explained?	All (1), some (0.5), or none (0) of the data types and structures of various modalities are explained
QC7	Were the feature extraction methods adequately described?	Feature extraction methods are adequately (1), partially (0.5), or poorly (0) described
QC8	Were the machine learning approaches adequately described?	Machine learning models and architectures are adequately (1), partially (0.5), or poorly (0) described
QC9	On a scale of 1–5, how reliable/effective was the machine learning approach?	Effectiveness, reliability and consistency of machine learning approach is well (5), partially (3), or poorly (0) justified through evaluation, analysis and baseline comparison
QC10	Was there a clear statement of findings?	Experimental findings are well (1), partially (0.5), or poorly (0) described
QC11	Were limitations to the results discussed?	Result limitations are well (1), partially (0.5), or poorly (0) identified
QC12	Was the study of value for research or practice?	Research methodology or outcomes well (1), partially (0.5), or poorly (0) contribute valuable findings or application

**Table 5:** Public datasets and respective data source categories.

Dataset	Description	Mental Health Disorders	Source Category
Distress Analysis Interview Corpus—Wizard of Oz (DAIC-WOZ) [228]	Video recordings and text transcriptions of interviews conducted by a virtual interviewer on individual participants (used in Audio-Visual Emotion Challenge (AVEC) 2014 [38], 2016 [47], 2017 [238], and 2019 [239])	Post-traumatic stress disorder (PTSD), depression, anxiety	AV
Turkish Audio-Visual Bipolar Disorder Corpus [230]	Video recordings of patients during follow-ups in a hospital	Bipolar disorder	AV
Engagement Arousal Self-Efficacy (EASE) [240]	Video recordings of individuals undergoing self-regulated tasks by interacting with a website	PTSD	AV
Well-being [241]	Video recordings of conversational interviews conducted by a computer science researcher	Depression, anxiety	AV
Emotional Audio-Textual Depression Corpus (EATD-Corpus) [73]	Audio responses and text transcripts extracted from student interviews conducted by a virtual interviewer through an application	Depression	AV
Reddit Self-Reported Depression Diagnosis Corpus (RSDD) [231]	Reddit posts of self-claimed and control users	Depression	SM
Self-Reported Mental Health Diagnosis Corpus (SMHD) [242]	Twitter posts of users with one or multiple mental health conditions and control users	ADHD, anxiety, autism, bipolar disorder, borderline personality disorder, depression, eating disorder, OCD, PTSD, schizophrenia, seasonal affective disorder	SM
Multi-modal Getty Image depression and emotion (MGID) dataset [106]	Textual and visual documents from Getty Image with equal amount of depressive and non-depressive samples	Depression	SM
Sina-Weibo suicidal dataset [243]	Sina microblog posts of suicidal and control users	Suicidal ideation	SM
Weibo User Depression Detection dataset (WU3D) [112]	Sina microblog posts of depressed candidates and control users, and user information such as nickname, gender and profile description	Depression	SM
Chinese Microblog depression dataset [244]	Sina microblog posts following the last posts of individuals who have committed suicide	Depression	SM
eRisk 2016 dataset [245]	Textual posts and comments of depressed and control users from Twitter, MTV’s A Thin Line (ATL) and Reddit	Depression	SM
eRisk 2018 dataset [246]	Textual posts and comments from Twitter, MTV’s A Thin Line (ATL) and Reddit	Depression, anorexia	SM
StudentLife [237]	Smartphone sensor data of students from a college	Mental wellbeing, stress, depression	SS
CrossCheck [205]	Smartphone sensor data of schizophrenia patients	Schizophrenia	SS
Student Suicidal Ideation and Depression Detection (StudentSADD [100]	Voice recordings and textual responses obtained using smartphone microphones and keyboards	Suicidal ideation, depression	AV, SS
BiAffect dataset [247]	Keyboard typing dynamics captured by a mobile application	Depression	SS
Tesserae dataset [248]	Smartphone and smartwatch sensor data, Bluetooth beacon signals, and Instagram and Twitter data of information workers	Mood, anxiety, stress	SS, WS, SM
CLPsych 2015 Shared Task dataset [249]	Twitter posts of users who publicly stated a diagnosis of depression or PTSD with corresponding control users of the same estimated gender with the closest estimated age	Depression, PTSD	SM
multiRedditDep dataset [128]	Reddit images posted by users who posted at least once in the */r/depression* forum	Depression	SM
Fitbit Bring-Your-Own-Device (BYOD) project by “All of Us” research program [250]	Fitbit data (e.g., steps, calories, and active duration), clinical assessments, demographics	Depression, anxiety	WS
PsycheNet dataset [138]	Social contagion-based dataset containing timelines of Twitter users and those with whom they maintain bidirectional friendships	Depression	SM
PsycheNet-G dataset [139]	Extends PsycheNet dataset [138] by incorporating users’ social interactions, including bidirectional replies, mentions, and quote-tweets	Depression	SM
Spanish Twitter Anorexia Nervosa (AN)-related dataset [251]	Tweets posted by users whom clinical experts identified to fall into categories of AN (at early and advanced stages of AN but do not undergo treatment), treatment, recovered, focused control (control users that used AN-related vocabulary), and random control	AN	SM
Audio-visual depressive language corpus (AViD-Corpus) [37]	Video clips of individuals performing PowerPoint-guided tasks, such as sustained vowel, loud vowel, and smiling vowel phonations, and speaking out loud while solving a task (used in AVEC 2013 [37])	Depression	AV
Existing call log dataset [222]	Call and text messaging logs and GPS data collected via mobile application and in-person demographic and mental wellbeing surveys	Mental wellbeing	SS
Speech dataset [252]	Audio recordings of individuals performing two speech tasks via an external web application and demographics obtained from recruitment platform, Prolific [253]	Anxiety	AV
Early Mental Health Uncovering (EMU) dataset [104]	Data gathered via a mobile application that collects sensor data (i.e., text and call logs, calendar logs, and GPS), Twitter posts, and audio samples from scripted and unscripted prompts and administers PHQ-9 and GAD-7 questionnaires and demographic (i.e., gender, age, and student status) questions	Depression, anxiety	SS, AV, SM
Depression Stereotype Threat Call and Text log subset (DepreST-CAT) [105]	Data gathered via modifying the EMU application [104] to collect additional demographic (i.e., gender, age, student status, history of depression treatment, and racial/ethnic identity) and COVID-19 related questions	Depression, anxiety	SS, AV, SM
D-vlog dataset [92]	YouTube videos with equal amounts of depressed and non-depressed vlogs	Depression	AV

AV: Audio and video recordings, SM: Social media, SS: Smartphone sensors, WS: Wearable sensors.

**Table 6:** Categories of modality features.

Modality	Category	Description	Examples
Audio	Voice	Characteristics of audio signals	Mel-frequency cepstral coefficients (MFCCs), pitch, energy, harmonic-to-noise ratio (HNR), zero-crossing rate (ZCR)
	Speech	Speech characteristics	Utterance, pause, articulation
	Representations	Extracted from model architectures applied onto audio samples or representations	Features extracted from specific layers of pre-trained deep SoundNet [259] network applied onto audio samples
	Derived	Derived from other features via computation methods or models	High-level features extracted from long short-term memory (LSTM) [260] model applied onto SoundNet representations to capture temporal information
Visual	Subject/object	Presence or features of a person or object	Face appearance, facial landmarks, upper body points
	Representations	Extracted from model architectures applied onto image frames or representations	Features extracted from specific layers of VGG-16 network [261] (pre-trained on ImageNet [262]) applied onto visual frames
	Emotion-related	Capture emotions associated with facial expressions or image sentiment	Facial action units (FAUs) corresponding to Ekman’s model of six emotions [263], i.e., anger, disgust, fear, joy, sadness, and surprise, or eight basic emotions [264] that additionally include trust, negative and positive
	Textual	Textual content or labels	Quotes in images identified via optical character recognition (OCR)
	Color-related	Color information	Hue, saturation, color
	Image metadata	Image characteristics and format	Width, height, presence of exchangeable image file format (exif) file
	Derived	Derived from other features via computation methods or models	Fisher vector (FV) encoding [265] of facial landmarks
Textual	Linguistic	Language in terms of choice of words and sentence structure	Pronouns, verbs, suicidal keywords
	Sentiment-related	Emotion and sentiment components extracted via sentiment analysis (SA) tools	Valence, arousal and dominance (VAD) ratings
	Semantic-related	Meaning of texts	Topics and categories describing text content
	Representations	Vector representations generated using language models	Features extracted from pre-trained Bidirectional Encoder Representations from Transformers (BERT) [266] applied onto texts
	Derived	Derived from other features via computation methods or models	Features extracted from LSTM with attention mechanism applied onto textual representations to emphasize significant words
Social media	Post metadata	Information associated with a social media post	Posting time, likes received
	User metadata	Information associated with a social media user account	Profile description and image, followers, followings
	Representations	Representations of social network and interactions with other users	Graph network representing each user using a node and connecting two users mutually following each other
	Derived	Derived from other features via aggregation or encoding	Number of posts made on the weekends
Smartphone sensor	Calls and messages	Relating to phone calls and text messaging	Frequency and duration of incoming/outgoing phone calls
	Physical mobility	Inferences from accelerometer, gyroscope, and GPS data	Walking duration, distance traveled
	Phone interactions	Accessing phone, applications, and keyboards	Duration of phone unlocks, frequency of using specific applications, keystroke transitions
	Ambient environment	Surrounding illumination and noise	Brightness, human conversations
	Connectivity	Connections with external devices and environment	Association events with WiFi access points, occurrences of nearby Bluetooth devices
	Representations	High-level representations of time series sensor data	Features extracted from transformer to capture temporal patterns
	Derived	Derived from low-level features via computation or aggregation	Average weekly visited location clusters, sleep duration estimated from phone being locked and being stationary in a dark environment at night
Wearable sensor	Physical mobility	Inferences related to physical motion and sleep	Number of steps, sleep duration and onset time
	Physiological	Physiological signals	Heart rate, skin temperature
	Representations	High-level representations of time series sensor data	Features extracted from LSTM applied onto heart rate signals
Demographics and Personalities	Demographic	Personal demographic information	Age, gender
	Personality	An individual’s personality	Big 5 personality scores

## Data Availability

Not applicable.

## Abbreviations

The following abbreviations are used in this manuscript:

AdaBoost	Adaptive Boosting
ADHD	Attention Deficit Hyperactivity Disorder
BDI	Beck Depression Inventory
CES-D	Center for Epidemiological Studies Depression Scale
CNN	Convolutional neural network
DNN	Deep neural network
DSM-V	Diagnostic and Statistical Manual of Mental Disorders, Fifth Edition
ED	Eating disorder
GAD-7	General Anxiety Disorder-7
GPS	Global Positioning System
GRU	Gated recurrent unit
HDRS	Hamilton Depression Rating Scale
LSTM	Long short-term memory
MDD	Major depressive disorder
MFCC	Mel frequency cepstral coefficients
MH	Mental health
ML	Machine learning
MLP	Multi-layer perceptron
MRI	Magnetic Resonance Imaging
MTL	Multi-task learning
NN	Neural network
OCD	Obsessive-compulsive disorder
PHQ-9	Patient Health Questionnaire-9
PTSD	Post-traumatic stress disorder
RF	Random forest
SLR	Systematic literature review
SVM	Support vector machine
XGBoost	Xtreme Gradient Boosting

## Appendix A. Existing Modality Features

**Table A1:** Audio features.

Features	Tools	Studies	Feature Category
Low-level descriptors: jitter, shimmer, amplitude, pitch perturbation quotients, Mel-frequency cepstral coefficients (MFCCs), Teager-energy cepstrum coefficients (TECCs) [320], Discrete Cosine Transform (DCT) coefficients	OpenSmile [267], COVAREP [321], YAAFE [322], Praat [323], Python libraries (pyAudioAnalysis [324], DisVoice [325]), My-Voice Analysis [326], Surfboard [327], librosa [328]	[12,48,51,72,74,78,81,87,88,90,91,92,94,97,99,101,104,107,108,184,192,195,196,197,198,199,211,214]	Voice
Existing acoustic feature sets: Interspeech 2010 Paralinguistics [329], Interspeech 2013 ComParE [330], extended Geneva Minimalistic Acoustic Parameter Set (eGeMAPS) [331])	OpenSmile [267]	[51,57,59,61,63,74,81,97,103,192,194,195,196,197,198]	Voice
Speech, pause, laughter, utterances, articulation, phonation, intent expressivity	Praat [323], DeepSpeech [332]	[12,48,79,107,184,193,203,211]	Speech
Vocal tract physiology features	N/A	[49]	Speech
Embeddings of audio samples	VGG-16 [261], VGGish [333], DeepSpeech [332], DenseNet [334], SoundNet [259], SincNet [335], Wav2Vec [336], sentence embedding model [337], HuBERT [338], convolutional neural network (CNN), bidirectional LSTM (BiLSTM), ResNet [308], graph temporal convolution neural network (GTCN) [339]	[59,60,67,72,74,80,86,89,93,96,100,136,174,195]	Representations
Graph features: average degree, clustering coefficient and shortest path, density, transitivity, diameter, local and global efficiency	Visibility graph (two data points visible to each other are connected with an edge)	[81]	Representations
Statistical descriptors of voice/speech features: mean, standard deviation, variance, extreme values, kurtosis, 1st and 99th percentiles, skewness, quartiles, interquartile range, range, total, duration rate, occurrences, coefficient of variation (CV)	Manual computation, histograms, DeepSpeech [332]	[12,55,56,91,92,99,107,193,197,214]	Derived
Bag-of-AudioWords (BoAW) representations of voice/speech features	openXBOW [340]	[59,74]	Representations, Derived
High-level representations of features/representations (capture spatial and temporal information)	Gated recurrent unit (GRU) [341], LSTM, BiLSTM, combination of CNN residual and LSTM-based encoder–decoder networks [75], time-distributed CNN (T-CNN), multi-scale temporal dilated convolution (MS-TDConv) blocks, denoising autoencoder	[61,65,67,73,75,77,87,94,100,199]	Representations, Derived
Session-level representations from segment-level features/representations	Simple concatenation, Fisher vector encoding, Gaussian Mixture Model (GMM)	[192,199,214]	Representations, Derived
Facial/body appearance, landmarks, eye gaze, head pose	OpenFace [269], OpenCV [270], Viola Jones’ face detector [342], CascadeObjectDetector function in MATLAB’s vision toolbox, Haar classifier [270], Gauss–Newton Deformable Part Model (GN-DPM) [343], OpenPose [271], ZFace [344], CNN [46], Faster-RCNN (Region CNN) [147], multilevel convolutional coarse-to-fine network cascade [345], Inception-ResNet-V2 [346], VGG-Face [68], DenseNet [334], Affectiva https://go.affectiva.com/affdex-for-market-research (accessed on 10 December 2023), DBFace https://github.com/dlunion/DBFace (accessed on 10 December 2023), FaceMesh https://developers.google.com/android/reference/com/google/mlkit/vision/facemesh/FaceMesh (accessed on 10 December 2023), dlib [347]	[25,53,55,56,68,79,80,83,91,92,94,98,99,101,108,174,193,199,201,203,220]	Subject/Object
Appearance coefficients of facial image and shape	Active Orientation Model (AOM) [348]	[50]	Subject/Object
Probability distribution of 365 common scenes	Places365-CNN [349]	[220]	Subject/Object

**Table A2:** Visual features.

Features	Tools	Studies	Feature Category
Feature descriptors: local binary patterns, Edge Orientation Histogram, Local Phase Quantization, Histogram of Oriented Gradients (HOG)	OpenFace [269]	[47,48,53,195]	Subject/Object, Derived
Geometric features: displacement, mean shape of chosen points, difference between coordinates of specific landmarks, Euclidean distance, angle between landmarks, angular orientation	Manual computation, subject-specific active appearance model (AMM), AFAR toolbox [350]	[47,51,54,56,70,79,83,91,99,195,198,203]	Subject/Object, Derived
Motion features: movement across video frames, range and speed of displacements (facial landmarks, eye gaze direction, eye open and close, head pose, upper body points)	3D convolutional layers on persons detected at frame-level, Motion history histogram (MHH) [351], feature dynamic history histogram (FDHH), residual network-based dynamic feature descriptor [75]	[52,53,68,75,147,193]	Subject/Object, Derived
Facial action units (FAUs), facial expressions	OpenFace [269], Face++ [352], FACET software [353], AU detection module of AFAR [350]	[25,79,91,99,194,196,198]	Subject/Object, Emotion-related
FAU features: occurrences, intensities, facial expressivity, peak expressivity, behavioral entropy	MHH, Modulation spectrum (MS), Fast Fourier transform (FFT)	[83,99,101,107,194,354]	Emotion-related, Derived
Emotion profiles (EPs)	SVM-based EP detector [355]	[101]	Emotion-related
Sentiment score	ResNeXt [356]	[186]	Emotion-related
Turbulence features capturing sudden erratic changes in behaviors	N/A	[192]	Derived
Deep visual representations from images or video frames	VGG-16 [261], VGG-Face [357], VGGNet [261], AlexNet [358], ResNet [308] ResNet-50 [359], ResNeXt [356], EfficientNet [360], InceptionResNetV2 [346], CNN, dense201 [195], self-supervised DINO (self-distillation with no labels) [361], GTCN [339], unsupervised Convolutional Auto-Encoder (CAE) (replaces autoencoder’s fully connected layer with CNN) [195]	[53,58,60,74,82,84,85,89,93,95,98,106,111,115,116,117,122,125,126,129,131,132,135,160,187,195,201,220]	Representations
High-level (frame-level) representations of low-level features (LLDs, facial landmarks, FAUs)	Stacked Denoising Autoencoders (SDAE) [306], DenseXception block-based CNN [221] (replace DenseNet’s convolution layer with Xception layer), CNN-LSTM, denoising autoencoder, LSTM-based multitask learning modality encoder [62], 3D convolutional layers, LSTM	[55,62,87,98,199,221]	Representations, Derived
Session-level representations from frame-level features/representations	Average of frame-level representations, Fisher vector (FV) encoding, improved FV coding [265], GMM, Temporal Attentive Pooling (TAP) [75]	[55,75,117,199]	Representations, Derived
Texts extracted from images	python-tesseract [362]	[25,126,128,140]	Textual
Image labels/tags	Deep CNN-based multi-label classifier [113], Contrastive Language Image Pre-training (CLIP) [363], Imagga [364] (CNN-based automatic tagging system)	[113,124,128,129]	Textual
Bag-of-Words (BoVW) features	Multi-scale Dense SIFT features (MSDF) [365]	[124,195]	Textual, Derived
Color distribution-cool, clear, and dominant colors, pixel intensities	Probabilistic Latent Semantic Analysis model [366] (assigns a color to each image pixel), cold color range [367], RGB histogram	[20,140,145,204,220]	Color-related
Brightness, saturation, hue, value, sharpness, contrast, correlation, energy, homogeneity	HSV (Hue, Saturation, color) [368] color model	[20,106,109,113,145,204,220]	Color-related
Statistical descriptors for each HSV distribution: quantiles, mean, variance, skewness, kurtosis	N/A	[145,204]	Color-related, Derived
Pleasure, arousal, and dominance	Compute from brightness and saturation values [276]	[220]	Emotion-related, Derived
Number of pixels, width, height, if image is modified (indicated via exif file)	N/A	[204]	Image metadata

**Table A3:** Textual features.

Features	Tools	Studies	Feature Category
Count of words: general, condition-specific (depressed, suicidal, eating disorder-related) keywords, emojis	N/A	[20,104,109,123,126,127,130,133,134,137,145,146,187,188,218,219]	Linguistic
Words referring to social processes (e.g., reference to family, friends, social affiliation), and psychological states (e.g., negative/positive emotions)	Linguistic Inquiry and Word Count (LIWC) [278], LIWC 2007 Spanish dictionary [369], Chinese Suicide Dictionary [370], Chinese LIWC [371], TextMind [372], Suite of Automatic Linguistic Analysis Tools (SALAT) [279]—Simple Natural Language Processing (SiNLP) [373]	[20,79,109,118,121,128,186,194,196,197,198,204,211,219,374]	Linguistic
Part-of-speech (POS) tags: adjectives, nouns, pronouns	Jieba [375], Natural Language Toolkit (NLTK) [280], TextBlob [376], spaCy, Penn Treebank [377], Empath [378]	[61,100,104,123,126,135,184,185,189,195,218,219]	Linguistic
Word count-related representations: Term Frequency–Inverse Document Frequency (TF-IDF), Bag of Words (BoW), n-grams, Term Frequency–Category Ratio (TF-CR) [379]	Word2Vec embeddings, language models	[115,116,118,124,128,130,140,143,144,148,185,186,188,198,217,374]	Linguistic, Representations
Readability metrics: Automated Readability Index (ARI), Simple Measure of Gobbledygook (SMOG), Coleman–Liau Index (CLI), Flesch reading ease, Gunning fog index, syllable count scores	Textstat [380]	[218,220]	Linguistic
Lexicon-based representations [381]	Depression domain lexicon [382], Chinese suicide dictionary [370]	[120,135,189]	Representations
Sentiment scores, valence, arousal, and dominance (VAD) ratings	NLTK [280], IBM Watson Tone Analyzer, Azure Text Analytics, Google NLP, NRC emotion lexicon [383], senti-py [384], Stanford NLP toolkit [281], Sentiment Analysis and Cognition Engine (SEANCE) [282], text SA API of Baidu Intelligent Cloud Platform [123], Valence Aware Dictionary and Sentiment Reasoner (VADER) [385], Chinese emotion lexicons DUTIR [386], Affective Norms for English Words ratings (ANEW) [283], EmoLex [387], SenticNet [388], Lasswell [389], AFINN SA tool [390], LabMT [391], text2emotion [392], BERT [266]	[20,54,61,86,110,115,118,119,121,123,126,127,128,130,132,133,137,143,144,145,146,148,184,185,186,188,194,196,197,198,218,219,374]	Sentiment-related
Happiness scores of emojis	Emoji sentiment scale [393]	[110]	Sentiment-related
Emotion transitions from love to joy, from love to anxiety/sorrow (inspired by [394])	Chinese emotion lexicons DUTIR [386]	[187]	Sentiment-related
Word representations	Global vectors for word representation (GloVe) [395], Word2Vec [396], FastText [397], Embeddings from Language Models (ELMo) [398], BERT [266], ALBERT [297], XLNet [285], bidirectional gated recurrent unit (BiGRU) [341], itwiki (Italian Wikipedia2Vec model), Spanish model [399], EmoBERTa [298] (incorporate linguistic and emotional information), MiniLM [400] (supports multiple languages), GPT [401], TextCNN [402], Bi-LSTM [294]	[49,60,65,67,69,72,73,77,78,81,82,87,88,90,95,96,97,98,100,106,111,112,113,116,122,125,128,129,131,135,136,138,142,145,147,148,185,186,187,201,214,218,308]	Semantic-related, Representations
Sentence representations	Paragraph Vector (PV) [284], Universal Sentence Encoder [403], Sentence-BERT [404]	[52,59,70,71,89,102,103,174,199]	Semantic-related, Representations
Topic modeling, topic-level features	Scikit-learn’s Latent Dirichlet Allocation module [405], Biterm Topic Model [406]	[20,43,114,118,119,126,130,134,136,137,146,185,188,194,217,219]	Semantic-related
Description categories	IBM Watson’s Natural Language Understanding tool (https://cloud.ibm.com/apidocs/natural-language-understanding#text-analytics-features (accessed on 10 December 2023))	[132]	Semantic-related
High-level representations from low-level features/representations (e.g., sentence-level from word-level, to capture sequential and/or significant information)	BiLSTM with an attention layer, stacked CNN and BiGRU with attention, summarization [119] using K-means clustering and BART [407], combination of LSTM with attention mechanism and CNN, BiGRU with attention	[73,95,97,119,136,145,159,201]	Representations, Derived
User-level representations from post-level representations	CNN-based triplet network [408] from existing Siamese network [409] (consider cosine similarities between post-level representations between each individual and others in the same and different target groups), LSTM with attention mechanism	[128,138]	Representations, Derived
Session-level representations from segment-level representations	Fisher vector encoding	[199]	Representations, Derived
Subject-level average, median, standard deviation of sentiment scores, representations, POS counts	N/A	[110,185,186]	Derived
Subject-level representations in conversation	Graph attention network—vertex as question/answer pair incorporating LeakyReLU on neighbors with respective attention coefficients, edge between adjacent questions	[97]	Representations, Derived

**Table A4:** Social media features.

Features	Tools	Studies	Feature Category
Posts distribution (original posts, posts with images, posts of specific emotions/sentiments)-frequency, time	N/A	[109,112,122,123,126,130,134,137,142,145,188,218,219]	Post metadata
Username, followers, followings, status/bio description, profile header and background images, location, time zone	N/A	[109,115,118,122,123,126,130,134,137,142,145,171,188,218,219]	User metadata
Likes, comments, hashtags, mentions, retweets (Twitter), favourites (Twitter)	N/A	[115,126,135,137,142,171,185,189]	Social interactions, Post metadata
Stressful periods with stress level and category (study, work, family, interpersonal relation, romantic relation, or self-cognition)	Algorithm [410] applied on users’ posting behaviors	[187]	Post metadata, Derived
Aggregate posting time by 4 seasons, 7 days of the week, 4 epochs of the day (morning, afternoon, evening, midnight), or specific times (daytime, sleep time, weekdays, weekends)	N/A	[125,130,135,186,188,189,219]	Post metadata, Derived
Encoding of numerical features	Categorize into quartiles (low, below average, average, high)	[115]	Representations, Derived
Social interaction graph-node: user-level representations concatenated from post-level representations, edge: actions of following, mentioning, replying to comments, quoting	node2vec [411], Ego-network [412]	[139,185]	Social interactions
Personalized graph-user-level node: user-level representations made up of property nodes, property node (individual), personal information, personality, mental health experience, post behavior, emotion expression and social interactions, user–user edge: mutual following-follower relationship, user-property edge: user’s characteristics	Attention mechanism to weigh property by contribution to individual’s mental health condition (user-property edge) and emotional influence (user–user edge)	[187]	Social interactions
Retweet network node: user-level representations, directed edge: tweets of a user is retweeted by the directed user	Clustering-based neighborhood recognition-form communities with densely connected nodes, expand communities using similarity with adjacent nodes	[141]	Representations

**Table A5:** Smartphone sensor features.

Features	Tools	Studies	Feature Category
Phone calls and text messages: frequency, duration, entropy	N/A	[104,105,149,155,156,159,161,162,166,169,170,171,175,190,205,206,209,222,223]	Calls and messages
Phone unlocks: frequency, duration	Manual computation, RAPIDS [413]-a tool for data pre-processing and biomarker computation	[99,149,150,155,156,158,160,161,162,166,167,171,176,190,205,206,208,212,374,171]	Phone interactions
Phone charge duration	N/A	[163]	Phone interactions
Running applications: type, frequency, duration of usage	N/A	[99,149,150,155,156,158,160,161,162,166,169,170,171,190,205,206,208,212,374]	Phone interactions
Activity states (e.g., walking, stationary, exercising, running, unknown): frequency, duration	Android activity recognition API, activity recognition model (LSTM-RNN [414], SVM), Google Activity Recognition Transition API (using gyroscope and accelerometer)	[150,152,154,160,163,169,170,176,177,190,205,206]	Physical mobility
Footsteps	API of mobile devices, Euclidean norm of accelerometer data	[154,169,170]	Physical mobility
Distance traveled, displacement from home, location variance and entropy, time spent at specific places, transitions	Manual computation, RAPIDS [413]	[99,150,151,153,154,155,158,160,161,162,165,166,175,176,177,200,205,206,208,209]	Physical mobility
Location cluster features: number of clusters, largest cluster as primary location, most and least visited clusters	DBSCAN clustering [415], Adaptive K-means clustering [416]	[150,151,153,154,160,165,176,177,205,208]	Physical mobility
Speed	Compute from GPS and/or accelerometer	[153,165,166,209]	Physical mobility
Intensity of action	Compute rotational momentum from GPS and gyroscope	[162]	Physical mobility
GPS sensor, calls and phone screen unlock features	RAPIDS [413]-a tool for data pre-processing and biomarker computation	[158,164]	Physical mobility, Calls and messages, Phone interactions
WiFi association events (when a smartphone is associated or dissociated with a nearby access point at a location’s WiFi network)	N/A	[153]	Connectivity
Occurrences of unique Bluetooth addresses, most/least frequently detected devices	N/A	[99,151,155,156,175]	Connectivity
Surrounding sound: amplitude, conversations, human/non-human voices	N/A	[150,163,166,205,206,207,208,209]	Ambient environment
Surrounding illuminance: amplitude, mean, variance, standard deviation	N/A	[99,163,190,205,208,209]	Ambient environment
Silent and noise episodes: count, sum, minimum decibels	Detect via intermittent samples until noise state changes	[166]	Ambient environment
Sleep duration, wake and sleep onset	Infer from ambient light, audio amplitude, activity state, and screen on/off	[150,160,161,167,169,170,175,176,206]	Derived, Physical mobility
Keystroke features: count, transitions, time between two consecutive keystrokes	N/A	[166,202]	Phone interactions
Time between two successive touch interactions (tap, long tap, touch)	N/A	[166]	Phone interactions
Day-level features	Statistical functions (mean, median, mode, standard deviation, interquartile range) at the day-level or day of the week (weekdays, weekends)	[151,152,154,156,159,163,164,170,176,206]	Derived
Epoch-level features	Statistical functions at partitions of a day-morning, afternoon, evening, night	[149,151,152,156,159,163,166,176,206]	Derived
Hour-level features	Statistical functions at each hour of the day	[208,209]	Derived
Week-level features	Statistical functions at the week-level, distance from weekly mean	[162,164]	Derived
Rhythm-related features: ultradian, circadian, and infradian rhythms, regularity index [417], periodicity based on time windows	Manual computation, Cosinor [418]-a rhythmic regression function	[151,152,153,155,157,158,176,207]	Derived
Degrees of complexity and irregularity	Shannon entropy of sensor features	[166]	Derived
Statistical, temporal and spectral time series features	Time Series Feature Extraction Library (TSFEL) [419]	[104,105]	Derived
High-level cluster-based features: cluster labels, likelihood scores, distance scores, transitions	Gaussian mixture model (GMM) [420], partition around methods (PAM) clustering model [421]	[208,209]	Derived
Network of social interactions and personal characteristics: node type corresponds to a modality/category (e.g., individual, personality traits, social status, physical health, well-being, mental health status)	Heterogeneous Information Network (HIN) [422]	[173]	Representations
Representations capturing important patterns across timestamps	Transformer encoder [295]	[179]	Representations

**Table A6:** Wearable sensor features.

Features	Tools	Studies	Feature Category
Duration and onset of sleep status (asleep, restless, awake, unknown), sleep efficiency, sleep debt	API of wristband	[149,151,155,156,164,171,180,181,182,191,374]	Physical mobility
Number of steps, active and sedentary bouts, floor climb	API of wristband	[150,151,155,156,164,171,179,180,181,182,191,374]	Physical mobility
Heart rate (HR), galvanic skin response (GSR), skin temperature (ST), electrodermal activity (EDA)	API of Wristband	[149,150,164,169,170,172,178,179,182,191]	Physiological
Outliers of systolic and diastolic periods: centering tendency, spreading degree, distribution shape and symmetry degree values from blood volume pressure	N/A	[178]	Physiological, Derived
Motion features from accelerometer data: acceleration, motion	N/A	[149]	Physical mobility
Heart rate variability (HRV), rapid eye movement, wake after sleep onset, metabolic equivalent for task (MET) for physical activity	API of Oura ring	[158]	Physiological, Physical mobility
High-level features from HR, GSR, and ST signals	CNN-LSTM	[215]	Representations
Basal metabolic rate (BMR) calories	API of wristband	[179,180]	Physiological

**Table A7:** Demographic and personality features.

Features	Tools	Studies	Feature Category
Gender, age, location	Sina microblog user account	[187]	Demographic
Gender, age, relationships, education levels	bBridge [423], big data platform for social multimedia analytics	[20]	Demographic
Age, gender	Age and gender lexica [424], M3-inference model [425] performs multimodal analysis on profile images, usernames, and descriptions on social media profiles	[121,143,144]	Demographic
Big 5 personality scores	IBM’s Personality Insights [426], BERT-MLP model [427] on textual content	[57,121,130,143,144,188]	Personality
Proportion of perfection and ruminant thinking-related words in textual content (inspired by [287])	Perfection and ruminant-thinking-related lexicons	[187]	Personality
Interpersonal sensitivity: amount of stressful periods associated with interpersonal relations	Algorithm [410] applied on users’ posting behaviors	[187]	Personality

## Appendix B. Existing Modality Fusion Techniques

**Table A8:** Modality fusion techniques.

Category	Method	Tools	Studies
Feature level	Concatenate into a single representation	N/A	[67,84,85,89,96,97,105,132,142,143,145,146,166,170,179,197,199,200,201,217]
Score/Decision level	Sum-rule, product-rule, max-rule, AND and OR operations, or majority voting on modality-level scores	N/A	[48,51,56,77,87,98,126,173,193,198,201]
	Weighted average or sum of modality-level scores	N/A	[51,68,147,198,200]
	Average confidence scores from lower-level prediction	N/A	[121]
	Combine predictions of individual modalities as inputs to secondary ML models	SVM, decision tree, random forest, novel ML models	[48,52,56,64,71,72,74,103,122,155,193]
	Hierarchical score/decision-level fusion	Weighted voting fusion network [428]	[122,195]
	Summation of question-level scores from rules enforced on modality-specific predictions	N/A	[88]
Model level	Map multiple features into a single vector	LSTM-based encoder–decoder network, LSTM-based neural network, BiLSTM, LSTM, fully connected layer, tensor fusion network	[46,59,75,80,86,95,187]
	Concatenate feature representations as a single input to learn high-level representations	Dense and fully connected layers with attention mechanisms, CNN, multi-head attention network, transformer [295], novel time-aware LSTM	[70,73,77,89,91,92,94,125,189,214]
	Learn shared representations from weighted modality-specific representations	Gated Multimodal Unit (GMU) [429], parallel attention model, attention layer, sparse MLP (mix vertical and horizontal information via weight sharing and sparse connection), multimodal encoder–decoder, multimodal factorized bilinear pooling (combines compact output features of multi-modal low-rank bilinear [430] and robustness of multi-modal compact bilinear [431]), multi-head intermodal attention fusion, transformer [295], feed-forward network, low-rank multimodal fusion network [432]	[62,65,67,76,93,100,102,106,113,117,131,135,136,142,143,144,174,218,433]
	Learn joint sparse representations	Dictionary learning	[20]
	Learn and fuse outputs from different modality-specific parts at fixed time steps	Cell-coupled LSTM with L-skip fusion mechanism	[101]
	Learn cross-modality representations that incorporate interactions between modalities	LXMERT [434], transformer encoder with cross-attention layers (representations of a modality as query and the other as key/value, and vice versa), memory fusion network [435]	[82,92,129]
	Horizontal and vertical kernels to capture patterns across different levels	CASER [309]	[170]

## Appendix C. Existing Machine Learning Models

**Table A9:** Machine learning models.

Category	Machine Learning Models	Application Method	Studies
Supervised learning	Linear regression, logistic regression, least absolute shrinkage and selection operator (Lasso) regularized linear regression [436], ElasticNet regression [437], stochastic gradient descent (SGD) regression, Gaussian staircase model, partial least square (PLS) [438] regression (useful for collinear features), generalized linear models	Learn relationship between features to predict continuous values (scores of assessment scales) or probabilities (correspond to output classes)	[20,43,49,53,55,68,70,99,104,105,126,130,134,140,150,154,163,164,167,175,179,182,188,200,211,212,213,219,222,223]
	SVM	Find a hyperplane that best fits features (regression) or divides features into classes (classification), secondary model in score-level fusion	[47,50,79,99,104,105,115,121,130,134,140,148,162,163,169,178,179,188,198,210,219,223]
	One class SVM [439]	Anomaly detection by treating outliers as points on the other side of hyperplane	[165]
	Three-step hierarchical logistic regression	Incremental inclusion of three feature groups in conventional logistic regression	[181]
	Discriminant functions-Naive Bayes, quadratic discriminant analysis (QDA), linear discriminant analysis (LDA), Gaussian naive Bayes	Determine class based on Bayesian probabilities, detect state changes	[12,99,104,140,148,152,163,222]
	Decision tree	Construct a tree that splits into leaf nodes based on feature	[99,134,140,148,164,178]
	Mixed-effect classification and regression trees-generalized linear mixed-effects model (GLMM) trees [440]	Capture interactions and nonlinearity among features while accounting for longitudinal structure	[191]
Neural network	Fully connected (FC) layers, multilayer perceptron (MLP), CNN, LSTM, BiLSTM, GRU, temporal convolutional network (TCN) [441] (with dilation for long sequences)-with activation function like Sigmoid, Softmax, ReLU, LeakyReLU, and GeLU	Predict scores of assessment scales (regression) or probability distribution over classes (classification)	[60,78,80,84,85,86,87,88,90,91,92,93,94,96,98,105,111,113,117,131,133,135,136,142,143,144,146,162,163,167,168,170,172,174,178,179,190,197,199,201,218,219,221,223,308]
	DCNN-DNN (combination of deep CNN and DNN), GCNN-LSTM (combination of gated convolutional neural network, which replaces a convolution block in CNN with a gated convolution block, and LSTM)	The latter neural network makes predictions based on high-level global features learned by the prior	[52,308]
	Cross-domain DNN with feature adaptive transformation and combination strategy (DNN-FATC)	Enhance detection in the target domain by transferring information from a heterogenous source domain	[109]
	Attention-based TCN	Classify features using relational classification attention [442]	[72]
	One-hot transformer (lower complexity than original sine and cosine functions)	Apply one-hot encoding on features for classification	[72]
	Transformer [295]	Apply self-attention across post-level representations, attention masking masks missing information	[129]
	Transformer-based sequence classification models-BERT, RoBERTa [296], XLNet [285], Informer [443] (for long sequences)	Perform classification using custom pre-trained tokenizers augmented with special tokens for tokenization	[121,179]
	Hierarchical attention network (HAN) [444]	Predict on user-level representations derived from stacked attention-based post-level representations, each made up of attention-based word-level representations	[128]
	LSTM-based encoder and decoder	Learn factorized joint distributions to generate modality-specific generative factors and multimodal discriminative factors to reconstruct unimodal inputs and predict labels respectively	[82]
	GRU-RNN as baseline model with FC layers as personalized model	Train baseline model using data from all samples and fine-tune personalized model on individual samples	[161]
	CNN-based triplet network [408]	Incorporate representations of homogeneous users	[138]
	Stacked graph convolutional network	Perform classification on heterogeneous graphs by learning embeddings, sorting graph nodes, and performing graph comparisons	[139]
	GRU-D (introduce decay rates in conventional GRU to control decay mechanism)	Learn feature-specific hidden decay rates from inputs	[171]
Ensemble learning	Random forest (RF) [300], eXtreme Gradient Boosting (XGBoost), AdaBoost [301], Gradient Boosted Regression Tree [302] (GDBT) (less sensitive to outliers and more robust to overfitting)	Predict based on numerical input features	[51,99,104,105,114,126,130,134,140,148,151,155,157,160,163,164,167,169,178,179,182,183,188,203,204,206,212,219,222,223]
	RF	Secondary model that predicts from regression scores and binary outputs of individual modality predictions	[71,81]
	Balanced RF [445] (RF on imbalanced data)	Aggregate predictions of ensemble on balanced down-sampled data	[209]
	XGBoost-based subject-specific hierarchical recall network	Deduce subject-level labels based on whether the output probability of XGBoost at a specific layer exceeds a predetermined threshold	[194]
	Stacked ensemble learning architecture	Obtain the first level of predictions from KNN, naive Bayes, Lasso regression, ridge regression, and SVM, then use them as features of a second-layer logistic regression	[123]
	Feature-stacking (a meta-learning approach) [303]	Use logistic regression as an L1 learner to combine predictions of weak L0 learners on different feature sets	[185]
	Greedy Ensembles of Weighted Extreme Learning Machines (GEWELMs), WELM [446] (weighted mapping for unbalanced class), Kernel ELM	ELM [447] as a building block that maps inputs to class-based outputs via least square regression	[63,127,192]
	Stacked ensemble classifier	Use MLP as meta learner to integrate outputs of CNN base learners	[126]
	Cost-sensitive boosting pruning trees-AdaBoost with pruned decision trees	Weighted pruning prunes redundant leaves to increase generalization and robustness	[137]
	Weighted voting model	Weight predictions of baseline ML models (DT, Naive Bayes, KNN, SVM, generalized linear models, GDBT) based on class probabilities and deduce final outcome from the highest weighted class	[140]
	Ensemble of SVM, DT, and naive Bayes	N/A	[89]
	Combination of personalized LSTM-based and RF models	Train personalized LSTM on hourly time series data (of another sample most similar to the sample of concern based on demographic characteristics and baseline MH states), and RF on statistical and cluster-based features	[208]
Multi-task learning	CNN	Train jointly to produce two output branches, regression score and probability distribution for classification	[61,62]
	LSTM-RNN, attention-based LSTM subnetwork, MLP with shared and task-specific layers	Train for depression prediction with emotion recognition as the secondary task	[46,106,132]
	LSTM with Swish [448] activation function (speeds up training with the advantages of linear and ReLU activation), GRU with FC layers, DNN with multi-task loss function	Perform both regression and classification simultaneously	[74,102,118,141,193]
	Multi-task FC layers	Train jointly to predict severity level and discrete probability distribution	[97]
	Multi-output support least-squares vector regression machines (m-SVR) [304]	Map multivariate inputs to a multivariate output space to predict several tasks	[207]
	2-layer MLP with shared and task-specific dense layers with dynamic weight tuning technique	Train to perform individual predictions for positive and control groups	[180]
	Bi-LSTM-based DNNs to provide auxiliary outputs into DNN for main output	Auxiliary outputs correspond to additional predictions to incorporate additional information	[176]
	DNN (FC layers with Softmax activation) for auxiliary and main outputs	Train DNNs individually on different feature combinations as individual tasks to obtain auxiliary losses for joint optimization function of main output	[145]
	Multi-task neural network with shared LSTM layer and two task-specific LSTM layers	Train to predict male and female samples individually	[70]
Others	Semi-supervised learning-ladder network classifier [305] of stacked noisy encoder and denoising autoencoder [306]	Reconstruct input using outputs of noisy encoder in the current layer and decoder from the previous layer, combine with MLP (inspired by [449])	[196]
	DMF [450], RESCAL [451], DEDICOM [452], HERec [453]	Perform recommender system [307] approach on features modeled using HIN	[173]
	Graphlets [454], colored graphlets [455], DeepWalk [456], Metapath2vec++ [457]	Perform node classification on features modeled using HIN	[173]
	Combination of DBSCAN and K-Means	Density-based clustering	[78]
	Clustering-based-KNN	Deduce predicted class through voting of K-nearest data	[140,163,166,178,212,223]
	Linear superimpose of modality-specific features	Learn fitting parameters (between 0 and 1) that adjust the proportions of modality-specific features in the final outcome	[83]
	Two-staged prediction with outlier detection	Baseline ML model (LR, SVM, KNN, DT, GBDT, AdaBoost, RF, Gaussian naive Bayes, LDA, QDA, DNN, CNN) performs day-level predictions, t-test detects outliers in first stage outputs	[163]
	Label association mechanism	Apply to one-hot vectors of predictions from modality-specific DNNs	[189]
	Isolation Forest (ISOFOR) [458], Local Outlier Factor (LOF) [459], Connectivity-Based Outlier Factor (COF) [460]	Unsupervised anomaly detection	[166]
	Similarity and threshold relative to the model of normality (MoN) (from the average of deep representations of training instances in respective target groups)	Deduce predicted class based on higher similarity with corresponding MoN	[85]
	Federated learning based on DNN	Train global model on all data and fine-tune the last layer locally	[168]
